# Effectiveness of different digital interventions on symptoms for children and adolescents with attention-deficit/hyperactivity disorder: a network meta-analysis

**DOI:** 10.3389/fpsyt.2026.1747368

**Published:** 2026-06-10

**Authors:** Jingqi Mei, Wenhua Zhang, Jie Bin

**Affiliations:** 1 Department of Nursing, College of Medicine, Hunan Normal University, Changsha, Hunan, China; 2 College of Physical Education and Health, Guangxi Normal University, Guilin, Guangxi, China; 3 Department of Nursing, The 921th Hospital of Joint Logistics Support Force of the Chinese People’s Liberation Army, Changsha, Hunan, China

**Keywords:** attention-deficit hyperactivity disorder, digital intervention, executive function, hyperactivity impulsivity, inattention, network meta-analysis

## Abstract

**Background:**

Attention-deficit/hyperactivity disorder (ADHD) in children and adolescents is often treated effectively with medication, though this approach requires careful monitoring for side effects and long-term use. Consequently, digital interventions are emerging as a convenient and acceptable supplementary or alternative treatment option.

**Objective:**

The present study aims to systematically compare the efficacy of various digital interventions on core symptoms in children and adolescents diagnosed with ADHD through a network meta-analysis.

**Method:**

A comprehensive literature search was performed in the following databases: PubMed, Embase, the Cochrane Central Register of Controlled Trials, and Web of Science—from their inception to August 21, 2025. Outcome measures comprise three continuous variables: inattentive symptoms, hyperactive-impulsive symptoms, and executive function. These measures are assessed through subjective behavioral ratings or objective neuropsychological testing. Literature screening was conducted using EndNote 21 software. The quality of the included studies was assessed with the Cochrane Risk of Bias Assessment Tool, and the network meta-analysis was performed using Stata 17.0 software. Effect sizes were pooled using the standardized mean difference (SMDs) with 95% confidence intervals (CIs). Given the diversity of digital interventions, the age range of study participants, and the complexity of the control groups, it was determined that random-effects models would be employed for all analyses.

**Results:**

Traditional meta-analyses a total of 37 randomized controlled trials involving 2,922 participants. results indicate that digital interventions yield significant improvements in inattention [SMD = -0.44, 95% CI (-0.62, -0.26)] and hyperactivity-impulsivity [SMD = -0.26, 95% CI (-0.41, -0.12)] compared to control conditions. Although digital interventions showed a trend toward improvement in executive function, this effect did not reach statistical significance [SMD = -0.41, 95% CI (-0.85, 0.02)]. Subgroup analysis revealed that, compared to the control group, significant improvements in inattention were observed in subgroups involving computer-based cognitive tasks, virtual reality technology, neurofeedback, subjective behavioral scoring, and objective neuropsychological testing (all *P* < 0.05). For hyperactive and impulsive behaviors, significant improvements were observed across all subgroups: computer-based cognitive tasks, mobile gaming, mobile video viewing, and subjective behavioral ratings (all *P* values < 0.05). Regarding executive function, a significant improvement was found in the mobile gaming subgroup (*P* < 0.05). The network meta-analysis based on subjective behavior assessment scales included 32 randomized controlled trials involving 2,726 participants. The results indicate that neurofeedback achieved the highest relative SUCRA value (79.4%) for improving inattentive symptoms. Computer-based cognitive tasks yielded the highest relative SUCRA value (76.1%) for improving hyperactive/impulsive behaviors. Mobile gaming applications demonstrated the highest relative SUCRA value (72.9%) for enhancing executive function.

**Conclusion:**

This systematic review and network meta-analysis found that multiple digital interventions demonstrate positive effects in improving core symptoms of ADHD in children and adolescents. Cumulative Rank Probability Results from a Network Meta-Analysis of Subjective Measurement Tools, neurofeedback demonstrated the most pronounced advantage in ameliorating attention deficit symptoms, computerized cognitive tasks showed the greatest potential for alleviating hyperactivity-impulsivity symptoms, while mobile gaming exhibited the strongest potential for enhancing executive function. Given methodological heterogeneity among existing studies and the limited number of relevant publications included in the analysis, these conclusions warrant cautious interpretation.

**Systematic Review Registration:**

https://www.crd.york.ac.uk/, identifier CRD420251126367.

## 1 Introduction

Attention-deficit/hyperactivity disorder (ADHD) is a persistent neurodevelopmental disorder, with symptoms that typically emerge in early childhood and may continue into adulthood (1). Research indicates that the global prevalence of ADHD among children is approximately 7.6%, with higher rates observed in males than in females (2). ADHD not only impairs individual quality of life but also contributes to increased healthcare utilization and generates broader socioeconomic consequences. Current clinical guidelines generally recommend a stepped-care approach for the treatment of children and adolescents diagnosed with ADHD. For individuals presenting with more severe symptoms, non-pharmacological interventions are typically implemented first, followed by the introduction of pharmacological treatment if necessary. Common pharmacologic agents include central nervous system stimulants (e.g., methylphenidate, dextroamphetamine) and non-stimulants (e.g., atomoxetine). These medications have been shown to significantly improve the core symptoms of ADHD in the short term (3). However, treatment responses vary among individuals, and long-term use may be associated with certain adverse effects, such as decreased appetite, sleep disturbances, and mood swings (4). Non-pharmacological interventions also play an important role in the management of ADHD in children and adolescents, particularly as complementary or alternative approaches to pharmacological treatment. Cognitive training is a method aimed at improving cognitive functioning through systematic exercises and tasks, with the goal of enhancing performance in areas such as attention, memory, and executive functions (5). Digital interventions refer to the use of electronic and mobile technologies to enhance individuals’ self-management of symptoms. These interventions encompass various modalities, including neurofeedback, computerized cognitive tasks or games, mobile device games or videos, and virtual reality technologies (6). Previous research has demonstrated that computerized cognitive training can effectively improve cognitive functioning and behavioral symptoms in children with ADHD (7). Mobile devices offer a low-cost and accessible platform for digital interventions. Research indicates that smartphone-based psychoeducational applications are more effective than paper-based materials in improving attention deficits and impulsive behaviors among individuals with ADHD, while also enhancing task completion rates (8). In recent years, virtual reality technology has been widely applied in research involving children and adolescents with ADHD. By simulating real-world environments and providing immersive experiences, this technology has opened up new possibilities for the diagnosis, treatment, and cognitive training of ADHD (9).

During the diagnosis and treatment of ADHD in children and adolescents, the selection of digital intervention tools is of critical importance. However, there is currently a lack of direct comparative studies examining the effectiveness of different digital intervention approaches. Traditional pairwise meta-analyses are limited to comparing only two interventions at a time. Consequently, when multiple interventions are available, this approach makes it difficult to scientifically determine which method is most effective. Network meta-analysis enables the simultaneous evaluation of multiple interventions within a single model by integrating both direct and indirect evidence, allowing for comparative rankings of their effectiveness. Therefore, this study aims to systematically compare the effects of various digital interventions—including neurofeedback, computer-based cognitive tasks/games, mobile device games/videos, and virtual reality—against multiple control conditions on improving attention deficits, hyperactive-impulsive behaviors, and executive functions in children and adolescents with ADHD aged 5 to 18 years through a network meta-analysis, in order to identify the most effective intervention approaches. Among them, the comparison conditions will be explicitly categorized as passive controls (e.g., usual care) and active controls (e.g., pharmacological interventions, placebo control, electromyography biofeedback, simulated feedback), thereby providing data support with greater reference value for evidence-based clinical practice.

## 2 Materials and methods

### 2.1 Protocol and registration

The design and reporting of this study adhered to the Preferred Reporting Items for Systematic Reviews and Meta-Analyses (PRISMA) guidelines (10). The PRISMA-NMA checklist is provided in Appendix 5. This study has been registered with the PROSPERO international prospective register of systematic reviews (registration number: CRD420251126367).

### 2.2 Search strategy

This study conducted a systematic search of the PubMed, Embase, Cochrane Central Register of Controlled Trials, and Web of Science databases. The search period covered from the inception of each database to August 21, 2025. Through a manual search of the references cited in the included articles and relevant systematic reviews, additional studies were identified. The search terms are provided in Appendix 1.

### 2.3 Selection criteria

Inclusion Criteria:

- Study Design: Randomized Controlled Trial.
- Research participants: Children and adolescents aged 5–18 years with a clinical diagnosis of ADHD.
- Intervention: Any form of digital cognitive training (e.g., neurofeedback, computer-based tasks/games, mobile games/apps, virtual reality technologies).
- The control conditions included passive controls (e.g., usual care) and active controls (e.g., pharmacotherapy, placebo, electromyography biofeedback, simulated feedback).
- Outcome measures: Must include at least one of the following explicitly measured outcome categories: subjective behavioral assessments or objective neuropsychological testing of attention deficit, hyperactivity/impulsivity, or executive function.

Exclusion Criteria:

- Non-randomized studies, literature reviews, study protocols, conference abstracts, and duplicate publications were excluded.
- Literature for which the full text cannot be obtained or data is incomplete.
- Interventions not involving numerical cognition training.

### 2.4 Data extraction

After completing the literature search, EndNote 21 was used to pre-screen articles that met the inclusion criteria. Two reviewers independently screened the titles and abstracts to identify studies relevant to the present review. Subsequently, two researchers independently assessed and determined the studies to be included, with any disagreements resolved by a third party.

To compare the effects of different digital interventions on symptoms of ADHD, the interventions in this study were categorized as follows: (i) neurofeedback; (ii) computer-based cognitive training, including computerized cognitive tasks and computer-based cognitive games; (iii) mobile-based cognitive training, including mobile games and video content; and (iv) virtual reality. The classification criteria and examples of each intervention type are provided in Appendix 4. Control Group: (i) usual care; (ii) pharmacotherapy; (iii) placebo control; (iv) electromyographic biofeedback; (v) simulated feedback. The following data were extracted from each included study by two reviewers using a pre-developed form: first author, country, sample characteristics (age and sample size), mode of digital intervention delivery (software used, frequency, duration per session, and intervention period), assessment tools, and outcome measures. In cases where information was missing from the included studies, the corresponding authors were contacted via email.

### 2.5 Bias risk assessment

This study adhered to the guidelines outlined in the Cochrane Handbook (11) to assess the risk of bias in included randomized controlled trials. The evaluation encompasses several critical domains, including random sequence generation, allocation concealment, blinding implementation, selective reporting, data integrity, and the presence of other potential sources of bias. During the assessment process, two researchers independently evaluated each criterion and classified the risk of bias as “high,” “low,” or “unclear.” In cases of disagreement between the two researchers, a third researcher was consulted to make the final determination, ensuring the accuracy and reliability of the assessment.

### 2.6 Data synthesis and analysis

Data preprocessing and analysis were conducted independently by two researchers. Microsoft Office Excel was used to preprocess the raw data. Results are presented as changes between endpoint and baseline values, with all outcomes converted to means and their corresponding standard deviations. If the standard deviation was not reported, it was calculated from the standard error, confidence intervals, or other available metrics. In cases where the sample size was not provided in the analysis table, this information was extracted from the descriptive statistics.

For continuous outcomes of subjective behavioral assessments or objective neuropsychological testing regarding inattention, hyperactivity-impulsivity, and executive function, report effect sizes as standardized mean differences (SMD) with 95% confidence intervals (CI). In accordance with the Cochrane Guidelines for Interventions in Systematic Reviews, the choice of statistical model was based on *a priori* considerations of clinical and methodological heterogeneity, rather than on statistical tests of heterogeneity. Given the inherent substantial diversity within this review—encompassing varied digital intervention formats, a broad age range of participants (5–18 years), and complex control group conditions—we predetermined the use of random-effects models for all primary analyses. Subsequent subgroup analyses were conducted to explore potential sources of heterogeneity. To assess the validity of the network meta-analysis, the following examinations were conducted: ① Consistency Test: Global Inconsistency Test: A p-value greater than 0.05 indicates good overall consistency of the network, supporting the use of a consistent model. Local Inconsistency Test: For closed loops formed within the network, the node-splitting method is employed. A p-value greater than 0.05 for the comparison between direct and indirect evidence suggests no significant inconsistency within the loop. ② Assessment of Transitivity: Prior to analysis, the basic characteristics of the included studies (e.g., age, diagnostic criteria, intervention duration, measurement tools) were compared. If the characteristics were similar across studies, the transitivity assumption was considered to hold, allowing for the conduct of a network meta-analysis. ③ Inconsistency Handling: If (*P* <.05), adopt an inconsistency model or conduct sensitivity analyses to explore potential sources of inconsistency. ④ Effect sizes are reported as SMD with 95% CI. A 95% CI that does not include 0 is considered indicative of a statistically significant difference. The relative effectiveness of interventions was assessed using the surface under the cumulative ranking curve (SUCRA) values, with higher SUCRA values indicating a greater likelihood of an intervention being the most effective. It is important to note that the SUCRA value only reflects the relative probability of an intervention being ranked as the best among those compared in the network, and does not indicate the magnitude of the treatment effect or its clinical significance. Therefore, interpretation of SUCRA rankings should be complemented by a comprehensive analysis of the standardized mean difference estimates and their confidence intervals. Given the inherent differences in measurement properties between subjective behavioral assessment scales and objective neuropsychological tests, we plan to conduct stratified network meta-analyses for these two categories of outcome measures, where data permit, to assess the potential influence of measurement tools on study conclusions.

## 3 Result

### 3.1 Study selection

**Figure 1:**
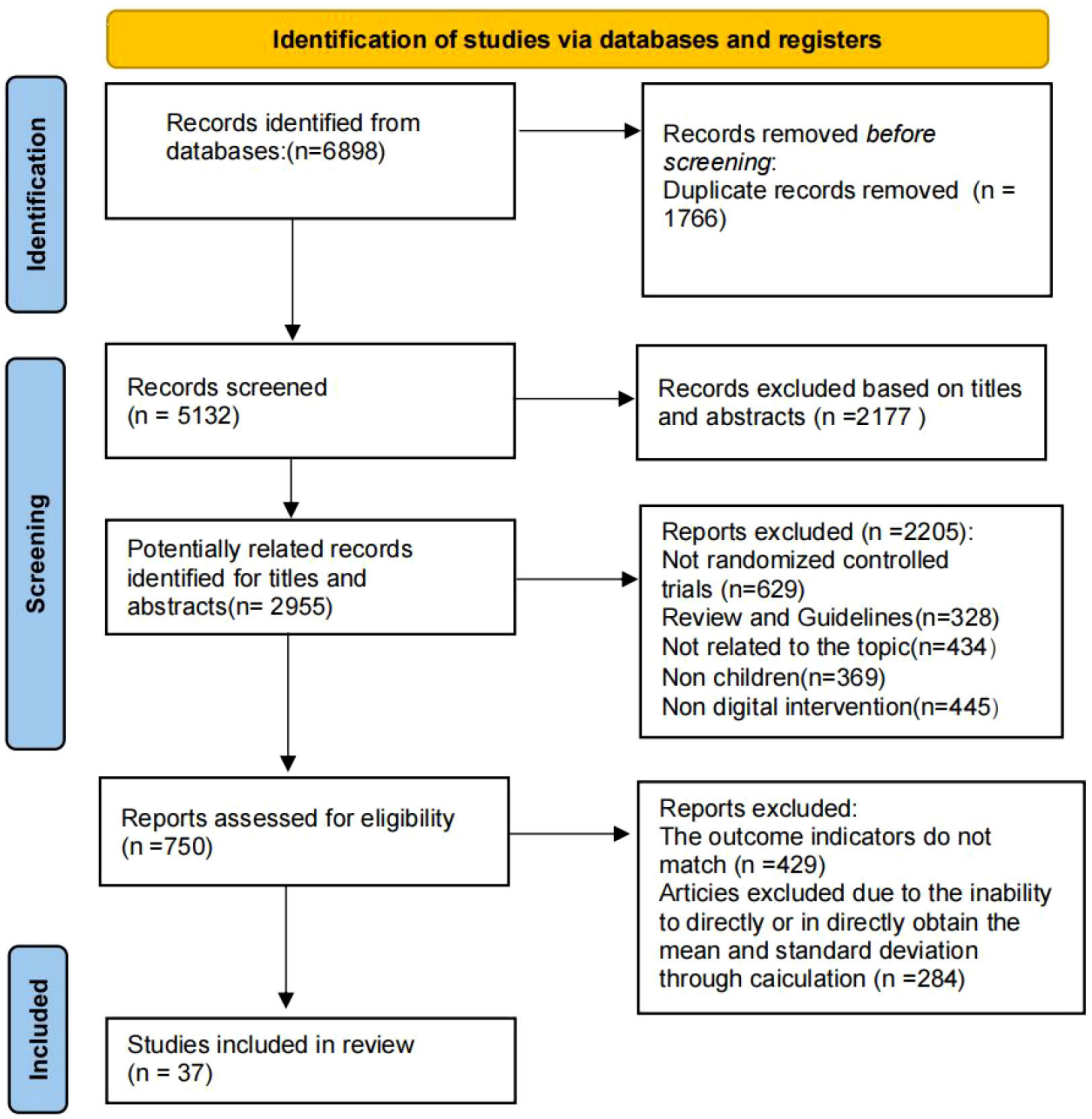
PRISMA study flow diagram (70).

A total of 6,898 records were initially identified through database searching. After removing duplicates using EndNote 21, 5,132 records remained. Following title and abstract screening, 2,177 records were excluded. Of the remaining 2,955 studies, 629 non-randomized controlled trials, 328 reviews and guidelines, 434 irrelevant studies, 369 studies not involving children and/or adolescents, 445 studies without digital interventions, 429 studies lacking relevant outcome indicators, and 284 studies with non-extractable data were excluded. Ultimately, 37 randomized controlled trials were included in this study, as illustrated in **Figure 1**.

### 3.2 Characteristics of included studies

This study included 37 articles involving a total of 2,922 participants. Among them, nine studies examined the effects of neurofeedback therapy, comprising 988 participants; seven studies assessed the effects of computerized cognitive tasks, involving 491 participants; eight studies evaluated the effects of mobile device games, encompassing 639 participants; four studies investigated the effects of virtual reality technology, including 167 participants; six studies explored the effects of computer game training, involving 368 participants; one intervention study compared the effects of mobile games versus mobile videos, including 50 participants; and two additional studies examined the effects of mobile device videos, comprising 219 participants. The characteristics of the included studies are summarized in **Table 1**.

**Table 1:** Characteristics of included studies.

Study	Country	n IGvsCG	Age IGvsCG	Interventions			Software	Comparison group	Assessment tools	Outcomes
				Type	Duration (w)	Day×min/w				
Dang (13)	China	60vs64	9.4 ± 1.4vs9.5 ± 1.7	Computer Cognitive Tasks	8	5×30	NR	Methylphenidate	ADHD-RS, BRIEF	a b c
Medina (14)	Spain	15vs14	9.2 ± 1.21vs9.71 ± 1.33	Mobile device games	12	3×15–20	KAD_SCL_01	Placebo-controlled	EDAH, BRIEF	a b c
Zhao (15)	China	40vs40	8.5 ± 1.5vs8.3 ± 1.1	Mobile device games	4	3×30	BrainFit	Usual Care	SNAP-IV, BRIEF	a b c
Zhu2025 (12)	China	53vs46	8.45 ± 1.08vs8.44 ± 1.29	Mobile device video	12	3×45-60	Ledongying	Usual Care	SNAP-IV	a b
Zheng & Shum (71)	China	14vs15vs21	5.02 ± 0.73vs4.89 ± 0.82	Mobile device video	5	3×10	NR	Usual Care	SNAP-IV, BRIEF	a b c
Azami (31)	Iran	12vs11	9.5 ± 1.44	Computer Cognitive Tasks	4	3×45	NR	Placebo-controlled	SNAP-IV, TOL	a b c
Bikic (23)	Denmark	35vs35	9.77 ± 1.97vs10.14 ± 1.52	Computer Cognitive Tasks	8	6×30	ACTIVATE™	Usual Care	ADHD-RS, BRIEF	a b c
Dovis (22)	Netherlands	31vs30	10.6 ± 1.4vs10.5 ± 1.3	Computer Cognitive Game;	5	5×35-50	Braingame Brian	Placebo-controlled	DBDRS, BRIEF	a b c
García-Redondo (32)	Spain	24vs20	11.83 ± 2.71vs11.83 ± 2.71	Mobile device games	14	2×10	Boogies Academy	Usual Care	EDAH	a b
Meyer (72)	USA	20vs20	9.84 ± 1.73vs10.82± 0.93	Computer Cognitive Tasks	4	5×13-15	NeuroScouting	Placebo-controlled	SNAP-IV	a b
Li (24)	China	30vs30	10.5 ± 1.2vs10.6 ± 1.3	Computer Cognitive Tasks	8	2×60	NeuroTrack	Usual Care	Conners 3, BRIEF	a b c
Qian (39)	China	18vs11	9.00 ± 1.50vs9.45 ± 1.29	Computer Cognitive Game;	8	3×30	CogoLand	Usual Care	ADHD-RS	a
Sudnawa (41)	Thailand	20vs20	8.4 ± 1.6vs9.0 ± 1.5	Computer Cognitive Game;	12	2–4×30	NR	Methylphenidate	VADRS	ab
Fan (42)	China	60vs60	9.63 ± 1.63vs9.11 ± 1.72	Mobile device video	8	1×45-60	DingTalk	Usual Care	SNAP-IV	a b
Weerdmeester (67)	Netherlands	37vs36	9.84 ± 1.71vs9.69 ± 1.79	Virtual Reality Technology	3	2×15	Dragon	Placebo-controlled	AVL	a b
Kirk (25)	Australia	28vs27	7.74 ± 0.93vs7.45 ± 0.93	Mobile device games	5	5×20	Tali Train	Placebo-controlled	SWAN, BRIEF	a b c
Smith (18)	USA	42vs38	7.6 ± 0.9vs7.2 ± 1.2	Computer Cognitive Tasks	15	4×30	NR	Usual Care	SNAP	a
Cho (33)	South Korea	10vs9	14--18	Virtual Reality Technology	2	4×20	NR	Usual Care	CPT	a
Wu (35)	China	50vs44	8.2 ± 1.31vs8.5 ± 1. 21	Computer Cognitive Tasks	8	6×25–40	ADHD executive function training	Placebo-controlled	ADHD-RS, BRIEF	a b c
S. Kim (34)	South Korea	20vs20	8--10	Virtual Reality Technology	6	2-3×30	NR	Usual Care	ATA	a
Bilan (68)	Spain	20vs21	9.41 ± 1.22vs9.38 ± 1.21	Mobile device games	12	3×15	KAD_SCL_01	Placebo-controlled	CPT	a
S.-C. Kim (30)	South Korea	16vs14	8.63 ± 1.86vs8.50 ± 1.29	Mobile device games	4	5×25	NeuroWorld DTx	Methylphenidate+Atomoxetine	K-ARS	a b
Kollins (20)	USA	180vs168	9.7 ± 1.3vs9.6 ± 1.3	Mobile device games	4	5×25	AKL-T01	Placebo-controlled	ADHD-RS	a
McDermott, Rose (36)	USA	21vs19	9.57 ± 1.34	Computer Cognitive Game;	6	3-4×15-20	Cogoland®	Usual Care	ADHD-RS	a
Steiner (21)	USA	13vs15	12.4 ± 0.9	Computer Cognitive Game;	16	2×45	BrainTrain	Usual Care	CRS, BRIEF	a b c
Bul (16)	Netherlands	88vs82	9.89 ± 1.28vs9.82 ± 1.24	Computer Cognitive Game;	10	3×65	Plan-It Commander	Usual Care	BRIEF	c
Bakhshayesh (37)	Germany	18vs17	9.6 ± 2.2vs9.1 ± 1.6	Neurofeedback	10	2–3×30	BioTrace	Electromyographic Biofeedback	FBB-HKS	a b
Lim (27)	Singapore	81vs82	8.7 ± 1.37vs8.6 ± 1.69	Neurofeedback	8	3×30	Cogoland	Usual Care	ADHD-RS	a
Ging-Jehli (38)	USA	78vs55	8 ± 1vs8 ± 1	Neurofeedback	12	3×25-45	EEGer Neurofeedback Software	Simulated feedback	Conners-3	a
Arnold (19)	USA	70vs50	8.52 ± 1.17vs8.7 ± 1.1	Neurofeedback	7	3×25-45	EEGer Neurofeedback Software	Simulated feedback	Conners-3	a b c
Lam (28)	UK	44vs44	10-18	Neurofeedback	4	4×10	AFNI	Simulated feedback	ADHD-RS	a
Purper (40)	France	90vs59	10.3 ± 1.8vs9.8 ± 1.8	Neurofeedback	12	4×30	Mensia Koala™	methylphenidate	ADHD-RS, BRIEF	a b c
Arnold (29)	USA	82vs55	8.51 ± 1.17vs8.67 ± 1.1	Neurofeedback	14	2-3×25-45	EEGer Neurofeedback Software	Simulated feedback	Conners-3	a b
Aggensteiner (73)	Switzerland	68vs64	8.6 ± 0.92vs8.57 ± 0.88	Neurofeedback	12	1-2×60	NEUROPRAX	Electromyographic Biofeedback	ADHD-RS	a b
Alegria (43)	UK	18vs13	14.11 ± 1.53vs13.62 ± 1.66	Neurofeedback	2	2×60	AFNI	Electromyographic Biofeedback	ADHD-RS	a b
Bioulac (74)	France	16vs19	9.5 ± 1.29vs8.8 ± 1.07	Virtual Reality Technology	6	2×30	NR	Usual Care	ADHD-RS	a b
Khanahmadi (26)	Iran	6vs6	5.33 ± 0.47vs5.67 ± 0.75	Mobile device games	8	NR	NR	Usual Care	PSQ	a b

w, weeks; a, inattention; b, hyperactivity and impulsivity; c, executive function; IG, Intervention Group CG, Control Group NR, No reports.

ADHD-RS, ADHD Rating Scale; BRIEF, Behavior Rating Inventory of Executive Function; EDAH, Escala de Evaluación del Déficit de Atención y la Hiperactividad; SNAP-IV, Swanson, Nolan, and Pelham–IV;TOL, Tower of London Test; DBDRS, Disruptive Behavior Disorder Rate Scale; Conners 3, Conners 3rd Edition; VADRS, Vanderbilt ADHD Diagnostic Rating Scale; AVL, ADHD VragenLijst; SWAN, Strengths and Weaknesses of ADHD-symptoms and Normal-behaviors; SNAP, Swanson, Nolan, and Pelham Rating Scale; MT, Multitasking Test; CPT, Continuous Performance Test; ATA, Advanced Test of Attention; K-ARS, Korean ADHD Rating Scale; CRS, Conners’ Rating Scale-Revised; FBB-HKS, German ADHD Rating Scale; PSQ, Pediatric Symptom Questionnaire;.

### 3.3 Risk of bias assessment results for studies included in the analysis

Using the Cochrane Risk of Bias tool to assess the 37 included studies, the majority were found to have a low risk of bias in terms of random sequence generation. 10 studies (12–21) reported methods of random sequence generation, such as computer-generated randomization, block randomization, and stratified randomization. Eleven studies (16, 18–20, 22–28) reported the use of allocation concealment, which was implemented through methods such as remote independent computer-generated randomization, sealed envelopes, or assignment by designated personnel. Eight studies (18–20, 22, 25, 28–30) explicitly reported using a double-blind design. Six studies (24, 26, 31–34) did not explicitly report attrition or missing data; all other studies provided details on participant attrition and follow-up. Twelve studies (12, 21, 24, 26, 32–39) did not provide trial registration numbers, whereas the remaining studies all provided trial registration numbers. Three studies (36–38, 40) conducted per-protocol (PP) analyses, while 11 studies (16, 18, 20, 22, 23, 25, 34, 35, 41–43) employed intention-to-treat (ITT) analyses. The remaining studies did not specify whether PP or ITT analysis was applied. The results of the risk of bias assessment are presented in Appendix 2.

### 3.4 Traditional meta-analysis

#### 3.4.1 Heterogeneity test and overall effect analysis

This Meta-analysis included a total of 37 studies. For inattention (n = 36), the heterogeneity test indicated substantial heterogeneity (*I^2^* = 79.6%, *P* < 0.001); The pooled effect size revealed that the experimental group demonstrated a significantly greater improvement in inattention compared to the control group [SMD = -0.44, 95% CI (-0.62, -0.26)]. For hyperactivity/impulsivity (n = 26), the heterogeneity test indicated substantial heterogeneity (*I^2^* = 55.2%, *P* < 0.001); The results showed that the experimental group was significantly superior to the control group in reducing hyperactivity/impulsivity [SMD = -0.26, 95% CI (-0.41, -0.12)]. For executive function (n = 14), the heterogeneity test indicated a high degree of heterogeneity (*I^2^* = 91.6%, *P* < 0.001); The analysis revealed that the experimental group did not demonstrate a significantly greater improvement in executive function compared to the control group [SMD = -0.41, 95% CI (-0.85, 0.02)]. Based on intervention measures and scale comparisons for cluster variables, further subgroup analyses were conducted to explore the sources of heterogeneity. Detailed results are provided in Appendix 3.

#### 3.4.2 Subgroup analysis results

##### 3.4.2.1 Subgroup analysis of inattentive

The results showed that computerized cognitive tasks [SMD = -0.44, 95% CI (-0.8, -0.07), *P* = 0.001, *I^2^* = 73.1%], virtual reality [SMD = -0.95, 95% CI (-1.86, -0.04), *P* < 0.001, *I^2^* = 85.3%], and neurofeedback [SMD = -0.43, 95% CI (-0.69, -0.18), *P* < 0.001, *I^2^* = 73%] each demonstrated significant effects. The effects of three interventions—mobile device games [SMD = -0.49, 95% CI (-1.06, 0.07), *P* < 0.001, *I^2^* = 89.3%], computerized cognitive games [SMD = -0.13, 95% CI (-0.45, 0.19), *P* = 0.294, *I^2^* = 19%], and mobile device videos [SMD = -0.40, 95% CI (-0.90, 0.09), *P* = 0.03, *I^2^* = 71.4%]—were not statistically significant.

The comparison between clusters showed that studies employing subjective behavioral ratings [SMD = -0.33, 95% CI (-0.50, -0.17), *P* < 0.001, *I^2^* = 73.4%] and those using objective neuropsychological tests [SMD = -1.64, 95% CI (-2.96, -0.32), *P* < 0.001, *I^2^* = 92%] both yielded statistically significant results.

##### 3.4.2.2 Subgroup Analysis of Hyperactivity and Impulsivity

Interventions: Results indicated that computer-based cognitive tasks demonstrated a significant effect [SMD = -0.43, 95% CI (-0.86, -0.01), *P* < 0.001, *I^2^* = 75.9%], as did mobile device games [SMD = -0.39, 95% CI (-0.74, -0.04), *P* = 0.061, *I^2^* = 50.1%] and mobile device videos [SMD = -0.37, 95% CI (-0.62, -0.12), *P* = 0.439, *I^2^* = 0%]. The effects of the three interventions were not statistically significant: virtual reality [SMD = 0.15, 95% CI (-0.71, 1.02), *P* = 0.034, *I^2^* = 77.6%], neurofeedback [SMD = -0.15, 95% CI (-0.31, 0.01), *P* = 0.741, *I^2^* = 0%], and computerized cognitive games [SMD = -0.19, 95% CI (-0.96, 0.57), *P* = 0.013, *I^2^* = 77.2%].

Cluster Comparison: The results showed that only the cluster comprising subjective behavioral ratings yielded a statistically significant effect [SMD = -0.27, 95% CI (-0.42, -0.11), *P* < 0.001, *I^2^* = 57%]. In contrast, the cluster of objective neuropsychological tests did not show a statistically significant effect [SMD = -0.25, 95% CI (-0.71, 0.21), *P* < 0.001, *I^2^* = 0%].

##### 3.4.2.3 Subgroup analysis of executive function

Intervention measures: Results indicate that only mobile device gaming (SMD = -0.37, 95% CI: -0.71 to -0.03, *P* = 0.244, *I^2^* = 28%) demonstrated a significant effect. For computer-based cognitive tasks [SMD = -1.00, 95% CI (-2.21, 0.22), *P* < 0.001, *I^2^* = 96.2%], neurofeedback [SMD = 0.17, 95% CI (-0.53, 0.87), *P* = 0.005, *I^2^* = 87.2%], computerized cognitive games [SMD = 0.05, 95% CI (-0.19, 0.29), *P* = 0.374, *I^2^* = 0%], and mobile video interventions [SMD = -0.11, 95% CI (-0.78, 0.55), *P* < 0.001, *I^2^* = 0%], the effects were not statistically significant.

The results of the cluster comparison showed that neither the studies using subjective behavioral ratings [SMD = -0.42, 95% CI (-0.87, 0.03), *P* < 0.001, *I^2^* = 92.2%] nor those employing objective neuropsychological tests [SMD = -0.28, 95% CI (-1.10, 0.54), *P* < 0.001, *I^2^* = 0%] yielded statistically significant findings.

### 3.5 Network meta-analysis

#### 3.5.1 Network evidence diagram

**Figure 2:**
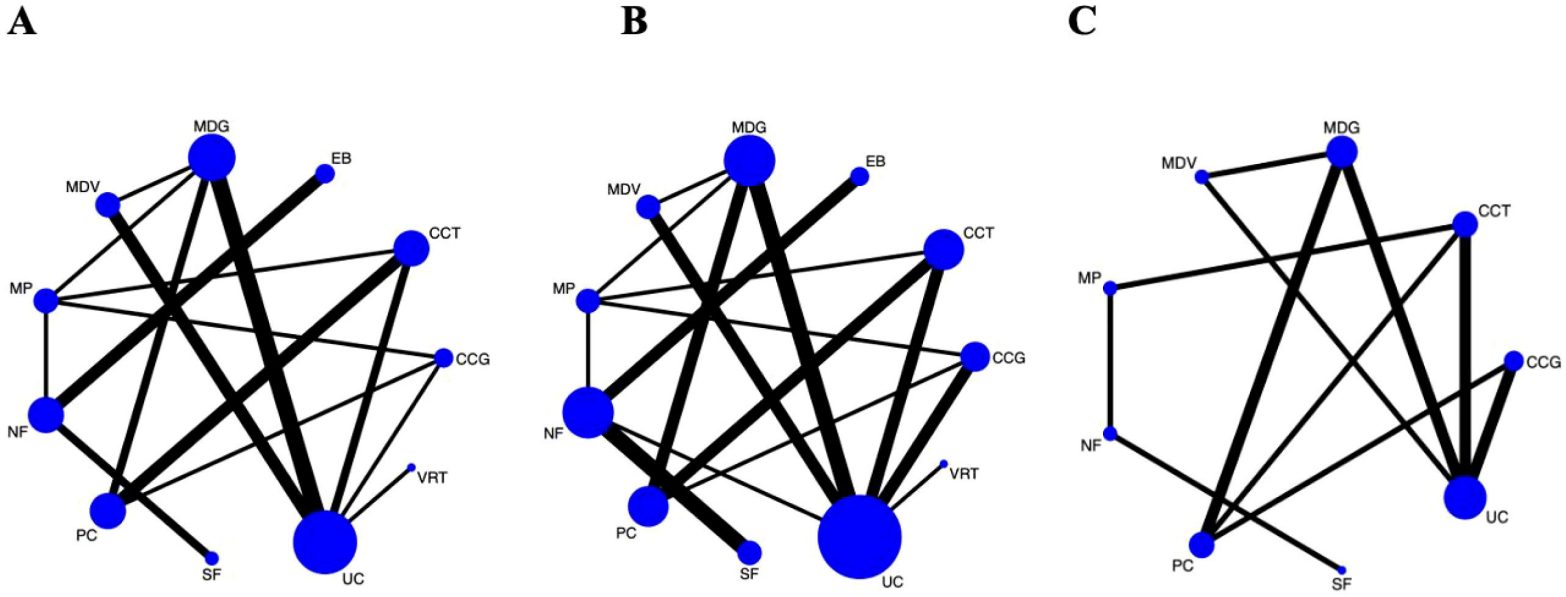
Network evidence diagram **(A)** inattention, **(B)** hyperactivity-impulsivity, **(C)** executive function. NF, Neurofeedback; CCT, Computer Cognitive Tasks; MDG, Mobile device games; VRT, Virtual Reality Technology; CCG, Computer Cognitive Game; MDV, Mobile device video; UC, Usual Care; SF, Simulated feedback; MP, Medicinal Products; EB, Electromyographic Biofeedback; PC, Placebo-controlled.

Building upon traditional meta-analyses, this network meta-analysis exclusively utilized subjective measurement tools due to the significant clinical heterogeneity introduced by combining subjective behavioral scales with objective neuropsychological tests. A total of 32 studies comprising 2,726 samples were included. In the relationships between interventions (**Figure 2**), dots represent intervention types, dot area denotes sample size, and connecting lines indicate direct comparisons between two interventions—with thicker lines signifying greater study numbers. The absence of a connecting line between two interventions indicates no direct comparison exists (44), Network meta-analyses may be employed for indirect comparisons. The control nodes in this study were not purely placebo controls; they incorporated control forms with explicit therapeutic activity, such as drug treatment and placebo controls. This directly elevated the relative effect size of standard care. To avoid potential over- or underestimation of effect sizes due to heterogeneity within the control group (including passive controls, placebo conditions, and drug treatments), which could influence SMD and SUCRA rankings, consequently, alongside routine care, active controls such as drug therapy and simulated feedback were incorporated to maintain methodological rigor.

#### 3.5.2 Inattention

The network graph (**Figure 2A**) illustrates the associations between various digital interventions and symptoms of inattention. A total of 32 studies involving 2579 participants were included to evaluate the effects of different interventions on inattention symptoms. Nine studies examined the efficacy of neurofeedback therapy, seven investigated the effects of computerized cognitive tasks, Seven evaluated the effectiveness of mobile device-based game interventions, A study exploring the role of virtual reality technology, five assessed the effectiveness of computer game training, two examined the impact of mobile device-based videos, and one intervention study simultaneously explored the combined effects of mobile device games and mobile device videos. The control group consisted of fourteen studies involving usual care, four studies involving simulated feedback intervention, seven studies involving placebo control intervention, four studies involving pharmacological intervention, and three studies involving an electromyographic biofeedback protocol. The p-value of 0.8929, being greater than 0.05, indicates no significant inconsistency across the entire network. Node-splitting analysis further revealed that both the direct and indirect comparisons yielded p-values greater than 0.05. Due to the low likelihood of local inconsistencies, a consistency model was adopted for the analysis. A network meta-analysis revealed that computer cognitive tasks [SMD = -0.52, 95% CI (-0.94, -0.09)], had a positive effect on inattention. Based on the SUCRA rankings presented in **Table 2**, neurofeedback emerged as the most effective digital intervention for children with ADHD-inattentive type, with the highest probability of being the optimal treatment, achieving a SUCRA value of 79.4%. The SUCRA scores for the other interventions were as follows: computer-based cognitive tasks (77.8%), mobile device video (64.2%), mobile device game (57.8%), placebo-controlled (51.9%), virtual reality technology (47.7%), electromyographic biofeedback (47.3%), computer cognitive game (43.9%), simulated feedback (37.6%), usual care (21.3%), and medicinal products (21.1%).

**Table 2:** Ranking of effects of different types of digital cognitive interventions on inattention, hyperactivity impulsivity, and executive function.

Intervention	Inattention			Hyperactive impulsivity			Executive function		
	SUCRA (%)	PrBest (%)	MeanRank	SUCRA (%)	PrBest (%)	MeanRank	SUCRA (%)	PrBest (%)	MeanRank
Computer cognitive task	77.8	20.9	3.2	76.1	24.0	3.4	70.4	24.4	3.4
Computer cognitive game	43.9	2.2	6.6	58.2	11.3	5.2	66.1	7.3	3.7
Mobile device game	57.8	3.9	5.2	54.5	4.7	5.6	72.9	26.4	3.2
Mobile device video	64.2	14.2	4.6	38.6	2.2	7.1	69.8	22.3	3.4
Virtual reality technology	47.7	17.9	6.2	2.1	—	10.8	—	—	—
Neurofeedback	79.4	29.9	3.1	75.3	22.9	3.5	9.5	—	8.2
Usual care	21.3	—	8.9	11.9	—	9.8	62.2	4.4	4.0
Placebo-controlled	51.9	3.1	5.8	52.9	3.2	5.7	71.1	15.3	3.3
Electromyographic biofeedback	47.3	6.4	6.3	59.9	14.6	5.0	—	—	—
Simulated feedback	37.6	1.4	7.2	56.7	10.7	5.3	4.2	0.0	8.7
Medicinal products	21.1	0.2	8.9	63.9	6.5	4.6	23.8	—	7.1

#### 3.5.3 Hyperactivity and impulsivity

**Figure 2B** presents a network diagram illustrating the associations between various digital interventions and impulsive behaviors. A total of 25 studies, comprising 1,698 participants, were included to evaluate the effects of different interventions on symptoms of hyperactivity-impulsivity. Six studies examined the effectiveness of neurofeedback therapy, six investigated the effects of computer-based cognitive tasks, and six assessed the impact of interventions using mobile device games. A study has examined the role of virtual reality technology, three evaluated the effectiveness of computer game training, and two examined the effects of mobile device videos. An additional intervention study investigated the combined effects of mobile device games and videos. The control group comprised ten studies involving usual care, two studies involving simulated feedback, six studies involving a placebo control, four studies involving medicinal products, and three studies involving an electromyographic biofeedback protocol. The p-value of 0.8878, which exceeds the 0.05 threshold, indicates no significant inconsistency across the overall network. Node-splitting analysis revealed p-values greater than 0.05 for both direct and indirect comparisons, suggesting a low probability of local inconsistency. Accordingly, the consistency model was employed for the analysis. A network meta-analysis revealed that both computer-based cognitive tasks (SMD = -0.76, 95% CI (-1.18, -0.35)], medicinal products [SMD = -0.66, 95% CI (-1.22, -0.11)], computer cognitive game [SMD = -0.60, 95% CI (-1.17, -0.03)], placebo-controlled [SMD = -0.56, 95% CI (-1.03, -0.09)] and mobile device games [SMD = -0.48, 95% CI (-0.89, -0.07)] exerted significant effects on hyperactivity-impulsivity symptoms. Ranking probabilities based on the SUCRA indicated that computer-based cognitive tasks were the most effective digital intervention for reducing hyperactivity-impulsivity symptoms in children with ADHD, with a SUCRA value of 76.1% (**Table 2**). In the SUCRA evaluation, the ranking of the other interventions was as follows: neurofeedback (75.3%), medicinal products (63.9%), electromyographic biofeedback (59.9%), computer cognitive game (58.2%), simulated feedback (56.7%), mobile device game (54.5%), placebo-controlled (52.9%), mobile device video (38.6%), usual care (11.9%) and virtual reality technology (2.1%).

#### 3.5.4 Executive function

**Figure 2C** presents a network graph illustrating the associations between various digital interventions and executive functions. This study synthesized 13 research projects involving 1,090 participants to evaluate the effects of different interventions on executive functioning. Of these, two studies examined the effectiveness of neurofeedback therapy, four investigated the effects of computer-based cognitive training, three explored the impact of mobile device games, three discussed the outcomes of computer-based game training, and one assessed the combined effects of mobile device games and mobile device videos. The control group comprised six studies involving usual care, one study involving simulated feedback, four studies involving a placebo control, and two studies involving medicinal products. The p-value of 0.6083, which exceeds the 0.05 threshold, indicates that there is no significant inconsistency across the entire network. Node-splitting analysis further revealed that all p-values for both direct and indirect comparisons were greater than 0.05, suggesting a low likelihood of local inconsistencies. Consequently, the consistency model was employed for the analysis. A network meta-analysis revealed that both computer-based cognitive tasks [SMD = -0.52, 95% CI (-0.93, -0.12)] and mobile device games [SMD = -0.53, 95% CI (-0.94, -0.12)] exerted significant effects on executive function. Based on the ranking of interventions in **Table 2**, Mobile device game had the highest probability of being the most effective digital intervention for improving executive function in children with ADHD, with a SUCRA value of 72.9%. The SUCRA values for the other interventions were as follows: placebo-controlled (71.1%), computer-based cognitive tasks (70.4%), mobile device videos (69.8%), computer-based cognitive games (66.1%), usual care (62.2%), medicinal products (23.8%), neurofeedback (9.5%) and Simulated feedback (4.2%).

### 3.6 Sensitivity analysis

Sensitivity analysis is a method used to evaluate the robustness of findings under specific assumptions. It involves adjusting key factors—such as inclusion criteria, study quality, attrition rates, or effect sizes—and subsequently re-conducting the meta-analysis. The results are then compared with the original findings to assess the stability of the conclusions. This study employed a leave-one-out sensitivity analysis to assess the influence of individual studies on the overall pooled effect size. As presented in Appendix 3, the recalculated effect sizes after omitting each study individually all fell within the 95% confidence interval of the overall effect size. These findings suggest that no single study had a disproportionate impact on the pooled estimate, indicating that the results are robust. This sensitivity analysis further supports and strengthens the credibility of the original meta-analytic conclusions.

### 3.7 Publication bias

The study employed funnel plots to assess publication bias for the outcome measures of inattention, hyperactivity-impulsivity, and executive function. The majority of study effects were symmetrically distributed around the vertical line at x=0, although a small number of studies showed scattered distribution. Overall, these findings suggest a low likelihood of publication bias; however, caution is warranted when interpreting the results. The risk of bias in the included studies is detailed in Appendix 3.

## 4 Discussion

This systematic review and network meta-analysis integrates traditional meta-analytic methods, SUCRA-based probability rankings, and subgroup analyses to systematically evaluate the comparative efficacy of various digital interventions in improving attention deficits, hyperactivity-impulsivity, and executive function.

The results of the traditional meta-analysis indicated that, overall, digital interventions were significantly more effective than control conditions in improving attention deficits [SMD = -0.29, 95% CI (-0.37, -0.22)] and hyperactivity-impulsivity [SMD = -0.25, 95% CI (-0.40, -0.10)]. This is consistent with existing research findings. A meta-analysis by Westwood et al (7) confirmed that computerized cognitive training significantly improves attention and impulse control in children with ADHD. Regarding executive function outcomes, the present study observed a trend toward improvement following digital intervention, though the results did not reach statistical significance. This finding diverges from some previous research, such as the meta-analysis conducted by Yan et al (45) which suggested that computerized executive function training can lead to significant improvements in executive functioning among adolescents with ADHD.

It is noteworthy that this study identified discrepancies between conventional and network meta-analyses regarding executive function. The pooled estimate for all digital interventions failed to reach statistical significance, whereas the network meta-analysis indicated potential efficacy for computer-based cognitive tasks and mobile devices. This discrepancy may be attributed to the conventional meta-analysis evaluating the combined effect of heterogeneous digital interventions, whereas the network meta-analysis permits specific comparisons between interventions. Consequently, this apparent inconsistency reflects differences in analytical methodology.

The subgroup analysis further elucidated the efficacy characteristics of digital interventions, providing complementary and confirmatory evidence to existing research. In the subgroup of participants with attention deficits, computerized cognitive tasks, virtual reality technology, and neurofeedback demonstrated significant effects, consistent with the findings of Kim et al (17) This convergence supports the value of targeted cognitive training and neuromodulatory digital interventions in ameliorating attention impairments. In contrast, computerized cognitive games and mobile device games/videos did not yield significant effects, possibly because such applications often emphasize entertainment over targeted training, and their highly stimulating sensory content may further distract individuals with attention difficulties (46). In the subgroup showing improvement in hyperactivity-impulsivity, computerized cognitive tasks and mobile device games/videos demonstrated significant effects. This finding aligns with the work of Selaskowski et al (8), suggesting that the portability and interactivity of mobile devices may enhance training engagement in children with ADHD and contribute to the reduction of hyperactive-impulsive behaviors through gamified behavioral constraints (47). In the subgroup exhibiting improvements in executive function, only mobile device games showed a significant effect—a relatively distinct finding that diverges from existing literature. One possible explanation is that executive function represents the most complex cognitive domain in ADHD, encompassing multiple subcomponents such as working memory, cognitive flexibility, and inhibitory control (48). The heterogeneity in measurement tools used to assess executive function across the studies included in this meta-analysis may be a key factor contributing to the inconsistency between our subgroup result and prior findings. In addition, studies have shown that individuals with ADHD are more susceptible to smartphone addiction, highlighting the need to be mindful of the potential risks associated with prolonged digital media exposure (49). Excessive use of electronic devices has been linked to increased screen time, poorer sleep quality, and reduced face-to-face social interactions—factors that may negatively affect emotional regulation and overall development in children with ADHD (50, 51). In the future, when promoting digital interventions, it is essential to emphasize the importance of regulating usage duration, encourage engagement in offline activities, and incorporate digital health literacy education into family guidance and clinical practice. Such approaches can help balance the benefits of intervention with the protection of children’s physical and mental well-being.

In conventional meta-analyses, all interventions are compared against a composite control group comprising both active controls (e.g., pharmacological treatments, electromyographic biofeedback) and passive controls (e.g., standard care). This pooling may dilute intervention effects and exacerbate heterogeneity. In contrast, network meta-analyses separate different control conditions into independent nodes, enabling indirect comparisons between interventions. This study clarifies that SUCRA values represent relative ranking probabilities, and a high SUCRA ranking does not necessarily imply statistically or clinically significant effects. For instance, the SUCRA value for executive function routine care is higher than that for pharmacological treatment, neurofeedback, and model feedback. This may stem from the limited number of studies examining executive function as an outcome, sparse network structures, and reliance on indirect comparisons via intermediary studies. Consequently, the findings reflect statistical relative rankings rather than clinical superiority. Therefore, SUCRA results should always be interpreted in conjunction with the corresponding effect size and its confidence interval. According to conventional benchmarks, a standardized mean difference (SMD) value of 0.2 indicates a small effect, 0.5 denotes a moderate effect, and 0.8 signifies a large effect (52). Taking the significant effect size for computer-based cognitive tasks on impulsivity (SMD = −0.76) as an example, although this effect size approaches the large effect level, careful consideration must be given to factors such as research methodology, sample size, and control group conditions when interpreting the findings. Moreover, the near-transfer effects observed in cognitive training may not necessarily translate into substantive improvements in everyday functioning (53); Within the context of network meta-analysis, heterogeneity among control groups must be addressed under the transmissibility assumption. This assumption requires that the distribution of potential effect modifiers be comparable across different control conditions (54).

In a network meta-analysis based on subjective behavioral assessment scales, distinct digital interventions demonstrated differentiated advantages in ameliorating core ADHD symptoms: neurofeedback technology proved most effective in improving inattentiveness (SUCRA = 79.4%), computerized cognitive tasks ranked first for reducing hyperactivity-impulsivity (SUCRA = 76.1%), while mobile gaming devices demonstrated the greatest efficacy in enhancing executive function (SUCRA = 72.9%). The efficacy of neurofeedback technology in improving inattentiveness may be attributed to its mechanism of directly training the brain’s self-regulatory capacity through real-time modulation of electroencephalographic activity (55). Such neural-level alterations are more readily generalized to everyday behavior, thereby being effectively captured in subjective behavioral assessment scales completed by parents or teachers (56). Wu et al (57) network meta-analysis similarly confirmed that most neurofeedback therapies outperformed placebo controls in ameliorating ADHD symptoms, supporting their potential value in managing childhood ADHD. Notably, neurofeedback intervention studies are relatively straightforward to conduct with sham-feedback controls, and their blinding protocols are typically more rigorous, which may further enhance the reliability of their evidence. the advantages of computerized cognitive tasks in improving hyperactivity and impulsivity align with previous research findings. Zhou et al. (58) indicated that cognitive training, behavioral therapy, and neurofeedback all significantly enhance inhibitory control in children with ADHD. Cheng et al (59) conducted a longitudinal neuroimaging study revealing that personalized computer-based cognitive training significantly reduced abnormal activity in the cerebellum and hippocampus of children with ADHD, while enhancing neural activity in the anterior cingulate cortex and lingual gyrus. These neuroplastic changes were closely associated with improvements in core symptoms. The advantages of mobile gaming in enhancing executive function align with the experiential learning characteristics of gamified interventions. Lee et al (60) demonstrated through a systematic review and meta-analysis grounded in experiential learning theory that gamified digital interventions significantly improve visuospatial short-term memory and working memory in children with ADHD, potentially representing a key pathway for executive function enhancement. Gamified design enhances children’s engagement and training adherence by providing immediate feedback, progressive challenges, and immersive experiences. However, the study also noted that improvements in inhibitory control and behavioral monitoring, as measured by the BRIEF scale, did not reach statistical significance, suggesting that gamified interventions require further exploration in these domains. Progressive challenges and immersive experiences bolstered children’s engagement and adherence to training. Nevertheless, the study also indicated that improvements in inhibitory control and behavioral monitoring, as measured by the BRIEF scale, failed to reach statistical significance. This suggests that the enhancement of executive function through gamified interventions may manifest more prominently at the cognitive level rather than in everyday behavioral domains. Gabarron et al (61) review similarly noted that while digital interventions demonstrate potential benefits, the quality of evidence remains generally low.

An In-Depth Examination of Discrepancies Between Subjective and Objective Measurement Outcomes (1) Neurofeedback directly trains the brain’s self-regulatory capacity by modulating electroencephalographic activity in real time. Such neural-level alterations are more readily generalized to everyday behavior, thereby becoming detectable by parents/teachers via subjective scales (19, 29, 37, 38). In contrast, the core advantage of virtual reality technology lies in enhancing information processing efficiency within specific contexts by creating immersive environments. This effect exhibits strong situational dependency and is more readily demonstrated in standardized, objective laboratory cognitive tasks (53, 62) (2). Subjective scales assess comprehensive behavioral performance within everyday environments and are more sensitive to clinically meaningful long-term transfer effects, representing the gold standard recognized by regulatory bodies (63). Objective testing captures the transient performance of specific cognitive processes under laboratory conditions, exhibiting greater sensitivity to direct near-transfer effects of training but not necessarily generalizing to everyday functioning (7, 64). Neurofeedback may produce broader behavioral improvements through sustained neural modulation, thereby demonstrating optimal performance on this measurement dimension (3). Objective tests administered automatically by computers eliminate expectancy bias, yielding purer results. Subjective scales are predominantly completed by potentially informed parents/teachers, readily introducing expectancy bias. Only 21.6% of RCTs in this study employed double-blind designs, suggesting subjective scale outcomes may overestimate true effects (65). The SUCRA rankings in this study should be interpreted as relative efficacy based on clinical ratings and require cautious interpretation in conjunction with the sensitivity of the measurement tools (4). Certain objective testing tasks bore a high degree of similarity to intervention tasks, resulting in assessment outcomes reflecting task proficiency rather than fundamental improvements in core cognitive abilities (66). Subjective scales assessing broad daily behaviors did not exhibit this issue. It is noteworthy that the number of objective neuropsychological tests included in this study was extremely limited—only five (31, 33, 34, 67, 68) and exhibited high heterogeneity. This constrained the feasibility of using them as primary outcome measures in our network meta-analysis.

## 5 Strengths and limitations

(1) Outcome measures were limited to attention deficit, hyperactivity/impulsivity, and executive function, excluding other relevant domains. Although standardized mean differences were used to combine effect sizes, there remained variability in the cognitive assessment tools employed across studies (2). The age range of participants was broad (5–18 years), which, while enhancing statistical power, introduced clinical heterogeneity. Given the substantial differences between children and adolescents in cognitive development, neuroplasticity, executive function, and intervention adherence, subsequent studies should incorporate age-stratified designs to further examine the moderating effects of age (3). Only eight of the included studies adopted a double-blind design, a design that is less common in digital intervention trials due to the difficulty of implementing participant blinding. Previous meta-analyses have indicated that objective outcome measures and blinded assessors help reduce the risk of bias (69), However, the number of double-blind trials is limited; therefore, interpretations of the effect size should be approached with caution (4). Although strict selection criteria were applied, the included studies exhibited heterogeneity in terms of device brands, intervention protocols, and frequency. Future research should further investigate the sources of this variation (5). For outcome measures with limited studies incorporating executive function, network structures tend to be sparse. In such circumstances, high SUCRA values for certain control groups (e.g., standard care) do not necessarily indicate clinical efficacy but may represent statistical artefacts arising from indirect comparisons via intermediate nodes. Increased uncertainty can inflate relative rankings. Consequently, SUCRA probabilities must be interpreted alongside direct effect estimates and their confidence intervals (6). The observed discrepancy between subjective behavioral ratings and objective neuropsychological tests may reflect their differing constructs. Objective tasks are more sensitive to near transfer effects, whereas subjective scales capture distant transfer effects. Furthermore, only a minority of included trials employed double-blind designs, rendering subjective scoring susceptible to expectation bias from unblinded parents or teachers.

## 6 Conclusion

This study, through systematic review and network meta-analysis, found that multiple digital interventions demonstrate positive effects in improving core symptoms of ADHD in children and adolescents. Based on cumulative ranked probability results, neurofeedback holds the greatest advantage in improving inattentive symptoms, computerized cognitive tasks demonstrate the greatest potential in alleviating symptoms of hyperactive impulsivity. mobile games demonstrate the greatest potential in alleviating executive function difficulties. These should become key areas of focus for future intervention programs. However, although the overall traditional meta-analysis did not reveal statistically significant improvements in executive function following digital interventions, the indirect comparison results from the network meta-analysis suggest a potential trend indicating that computerized cognitive tasks and mobile device gamesmay offer superior benefits over other interventions in enhancing executive function. Given the methodological heterogeneity among existing studies and the limited number of relevant publications included in the analysis, this finding should be interpreted with caution. Future research requires additional large-scale, multicentre, high-quality randomized controlled trials to further validate these findings, particularly regarding the long-term effects on core measures such as executive function. This would provide more robust evidence-based guidance for clinical practice.

## Data Availability

The original contributions presented in the study are included in the article/**Supplementary Material**. Further inquiries can be directed to the corresponding author.
