# Supplementary material for: Effectiveness of different digital interventions on symptoms for children and adolescents with attention-deficit/hyperactivity disorder: a network meta-analysis
Source: Front Psychiatry. 2026 Jun 10;17:1747368. doi: 10.3389/fpsyt.2026.1747368 (PMC13292252; doi:10.3389/fpsyt.2026.1747368)
Supplement: Supplementary file 1 [file SupplementaryFile1.docx]

Supplementary Material

1. **Supplementary Tables**

**Supplementary Table S1.** PRISMA NMA Checklist of Items to Include When Reporting A Systematic Review Involving a Network Meta-analysis.

| **Section/Topic** | **Item #** | **Checklist Item** | **Reported on Page #** |
| --- | --- | --- | --- |
| **TITLE** |  |  |  |
| Title | 1 | Identify the report as a systematic review *incorporating a network meta-analysis (or related form of meta-analysis).* | **Title** |
|  |  |  |  |
| **ABSTRACT** |  |  |  |
| Structured summary | 2 | Provide a structured summary including, as applicable:  **Background:** main objectives  **Methods:** data sources; study eligibility criteria, participants, and interventions; study appraisal; and *synthesis methods, such as network meta-analysis.*  **Results:** number of studies and participants identified; summary estimates with corresponding confidence/credible intervals; *treatment rankings may also be discussed. Authors may choose to summarize pairwise comparisons against a chosen treatment included in their analyses for brevity.*  **Discussion/Conclusions:** limitations; conclusions and implications of findings.  **Other:** primary source of funding; systematic review registration number with registry name. | **Abstract** |
|  |  |  |  |
| **INTRODUCTION** |  |  |  |
| Rationale | 3 | Describe the rationale for the review in the context of what is already known*, including mention of why a network meta-analysis has been conducted.* | **1 Introduction** |
| Objectives | 4 | Provide an explicit statement of questions being addressed, with reference to participants, interventions, comparisons, outcomes, and study design (PICOS). | **1 Introduction** |
|  |  |  |  |
| **METHODS** |  |  |  |
| Protocol and registration | 5 | Indicate whether a review protocol exists and if and where it can be accessed (e.g., Web address); and, if available, provide registration information, including registration number. | **2.1 Protocol and registration** |
| Eligibility criteria | 6 | Specify study characteristics (e.g., PICOS, length of follow-up) and report characteristics (e.g., years considered, language, publication status) used as criteria for eligibility, giving rationale. *Clearly describe eligible treatments included in the treatment network, and note whether any have been clustered or merged into the same node (with justification).* | **2.3 Selection Criteria** |
| Information sources | 7 | Describe all information sources (e.g., databases with dates of coverage, contact with study authors to identify additional studies) in the search and date last searched. | **2.2 Search Strategy** |
| Search | 8 | Present full electronic search strategy for at least one database, including any limits used, such that it could be repeated. | **2.2 Search Strategy** |
| Study selection | 9 | State the process for selecting studies (i.e., screening, eligibility, included in systematic review, and, if applicable, included in the meta-analysis). | **2.4 Data extraction** |
| Data collection process | 10 | Describe method of data extraction from reports (e.g., piloted forms, independently, in duplicate) and any processes for obtaining and confirming data from investigators. | **2.4 Data extraction** |
| Data items | 11 | List and define all variables for which data were sought (e.g., PICOS, funding sources) and any assumptions and simplifications made. | **2.4 Data extraction** |
| **Geometry of the network** | **S1** | Describe methods used to explore the geometry of the treatment network under study and potential biases related to it. This should include how the evidence base has been graphically summarized for presentation, and what characteristics were compiled and used to describe the evidence base to readers. | **2.6 Data synthesis and analysis** |
| Risk of bias within individual studies | 12 | Describe methods used for assessing risk of bias of individual studies (including specification of whether this was done at the study or outcome level), and how this information is to be used in any data synthesis. | **2.5 Bias Risk Assessment** |
| Summary measures | 13 | State the principal summary measures (e.g., risk ratio, difference in means). *Also describe the use of additional summary measures assessed, such as treatment rankings and surface under the cumulative ranking curve (SUCRA) values, as well as modified approaches used to present summary findings from meta-analyses.* | **2.6 Data synthesis and analysis** |
| Planned methods of analysis | 14 | Describe the methods of handling data and combining results of studies for each network meta-analysis. This should include, but not be limited to:   - *Handling of multi-arm trials;* - *Selection of variance structure;* - *Selection of prior distributions in Bayesian analyses; and* - *Assessment of model fit.* | **2.6 Data synthesis and analysis** |
| **Assessment of Inconsistency** | **S2** | Describe the statistical methods used to evaluate the agreement of direct and indirect evidence in the treatment network(s) studied. Describe efforts taken to address its presence when found. | **2.6 Data synthesis and analysis** |
| Risk of bias across studies | 15 | Specify any assessment of risk of bias that may affect the cumulative evidence (e.g., publication bias, selective reporting within studies). | **2.5 Bias Risk Assessment** |
| Additional analyses | 16 | Describe methods of additional analyses if done, indicating which were pre-specified. This may include, but not be limited to, the following:   - Sensitivity or subgroup analyses; - Meta-regression analyses; - *Alternative formulations of the treatment network; and* - *Use of alternative prior distributions for Bayesian analyses (if applicable).* | **2.6 Data synthesis and analysis** |
|  |  |  |  |
| **RESULTS†** |  |  |  |
| Study selection | 17 | Give numbers of studies screened, assessed for eligibility, and included in the review, with reasons for exclusions at each stage, ideally with a flow diagram. | **3.1 Study selection** |
| **Presentation of network structure** | **S3** | Provide a network graph of the included studies to enable visualization of the geometry of the treatment network. | **3.5.1 Network Evidence Diagram** |
| **Summary of network geometry** | **S4** | Provide a brief overview of characteristics of the treatment network. This may include commentary on the abundance of trials and randomized patients for the different interventions and pairwise comparisons in the network, gaps of evidence in the treatment network, and potential biases reflected by the network structure. | **3.5.1 Network Evidence Diagram** |
| Study characteristics | 18 | For each study, present characteristics for which data were extracted (e.g., study size, PICOS, follow-up period) and provide the citations. | **3.2 Characteristics of included studies** |
| Risk of bias within studies | 19 | Present data on risk of bias of each study and, if available, any outcome level assessment. | **3.3 Risk of bias assessment results for studies included in the analysis** |
| Results of individual studies | 20 | For all outcomes considered (benefits or harms), present, for each study: 1) simple summary data for each intervention group, and 2) effect estimates and confidence intervals. *Modified approaches may be needed to deal with information from larger networks.* | **3.4 Traditional Meta-Analysis**  **3.5 Network Meta-Analysis** |
| Synthesis of results | 21 | Present results of each meta-analysis done, including confidence/credible intervals. *In larger networks, authors may focus on comparisons versus a particular comparator (e.g. placebo or standard care), with full findings presented in an appendix. League tables and forest plots may be considered to summarize pairwise comparisons.* If additional summary measures were explored (such as treatment rankings), these should also be presented. | **3.4 Traditional Meta-Analysis**  **3.5 Network Meta-Analysis** |
| **Exploration for inconsistency** | **S5** | Describe results from investigations of inconsistency. This may include such information as measures of model fit to compare consistency and inconsistency models, *P* values from statistical tests, or summary of inconsistency estimates from different parts of the treatment network. | **3.5 Network Meta-Analysis** |
| Risk of bias across studies | 22 | Present results of any assessment of risk of bias across studies for the evidence base being studied. | **3.3 Risk of bias assessment results for studies included in the analysis** |
| Results of additional analyses | 23 | Give results of additional analyses, if done (e.g., sensitivity or subgroup analyses, meta-regression analyses*, alternative network geometries studied, alternative choice of prior distributions for Bayesian analyses,* and so forth). | **3.4.2 Subgroup Analysis Results**  **3.6 Sensitivity Analysis**  **3.7 Publication bias** |
|  |  |  |  |
| **DISCUSSION** |  |  |  |
| Summary of evidence | 24 | Summarize the main findings, including the strength of evidence for each main outcome; consider their relevance to key groups (e.g., healthcare providers, users, and policy-makers). | 1. **Discussion** |
| Limitations | 25 | Discuss limitations at study and outcome level (e.g., risk of bias), and at review level (e.g., incomplete retrieval of identified research, reporting bias). *Comment on the validity of the assumptions, such as transitivity and consistency. Comment on any concerns regarding network geometry (e.g., avoidance of certain comparisons).* | **5 Strengths and limitations** |
| Conclusions | 26 | Provide a general interpretation of the results in the context of other evidence, and implications for future research. | **6 Conclusion** |
|  |  |  |  |
| **FUNDING** |  |  |  |
| Funding | 27 | Describe sources of funding for the systematic review and other support (e.g., supply of data); role of funders for the systematic review. This should also include information regarding whether funding has been received from manufacturers of treatments in the network and/or whether some of the authors are content experts with professional conflicts of interest that could affect use of treatments in the network. | Funding |

PICOS = population, intervention, comparators, outcomes, study design.

* Text in italics indicateS wording specific to reporting of network meta-analyses that has been added to guidance from the PRISMA statement.

† Authors may wish to plan for use of appendices to present all relevant information in full detail for items in this section.

# Supplementary Table S2. Search strategy in PubMed.

| Step | Search strategy |
| --- | --- |
| #1 | Search: (((((("Computers"[Mesh]) OR (Calculators, Programmable)) OR (Programmable Calculator)) OR (Hardware, Computer)) OR (Computer Hardware)) OR (Computers, Digital)) OR (Digital Computer |
| #2 | Search: (("Neurofeedback"[Mesh]) OR (Brainwave Biofeedback)) OR (Biofeedback, Brainwave)) OR (Brainwave Biofeedbacks)) OR (Alpha Biofeedback)) OR (Biofeedback, Alpha)) OR (Alpha Feedback)) OR (Feedback, Alpha)) OR (Brainwave Feedback)) OR (Feedback, Brainwave)) OR (EEG Feedback)) OR (Feedback, EEG)) OR (Electroencephalography Biofeedback)) OR (Electromyography Feedback) |
| #3 | Search:((("Virtual Reality"[Mesh]) ) OR (Reality, Virtual)) OR (Virtual Reality, Educational)) OR (Educational Virtual Realities)) OR (Reality, Educational Virtual)) OR (Virtual Realities, Educational)) OR (Virtual Reality, Instructional)) OR (Instructional Virtual Realities)) OR (Realities, Instructional Virtual) |
| #4 | Search: (("Smartphone"[Mesh]) OR (Smart Phone)) OR (Phones, Smart)) OR (Smart Phones) |
| #5 | Search:((("Computers, Handheld"[Mesh]) OR (Handheld Computer)) OR (Computers, Palmtop)) OR (Palmtop Computer)) OR (Computers, Palm-Top)) OR (Palm-Top Computer)) OR (Personal Digital Assistant)) OR (Digital Assistant, Personal)) OR (PDA Computer)) OR (PDA Computers)) OR (Palm Pilot)) OR (Pocket PC)) OR (Tablet Computers)) OR (Mobile Devices) |
| #6 | Search: ((("Video Games"[Mesh]) OR (Game, Video)) OR (Computer Games)) OR (Game, Computer) |
| #7 | Search:(("Wearable Electronic Devices"[Mesh]) OR (Device, Wearable Electronic)) OR (Wearable Electronic Device)) OR (Wearable Devices)) OR (Wearable Technology)) OR (Electronic Skin)) OR (Wearable Computer) |
| #8 | #1 OR #2 OR #3 OR #4 OR #5 OR #6 OR #7 |
| #9 | Search:((("Attention Deficit Disorder with Hyperactivity"[Mesh]) ) OR (ADHD)) OR (ADDH)) OR (Attention Deficit Disorders with Hyperactivity)) OR (Attention Deficit Hyperactivity Disorders)) OR (Attention Deficit Hyperactivity Disorder)) OR (Deficit-Hyperactivity Disorder, Attention)) OR (Disorder, Attention Deficit-Hyperactivity)) OR (Hyperkinetic Syndrome)) OR (Syndromes, Hyperkinetic)) OR (Attention Deficit Disorder)) OR (Disorder, Attention Deficit)) OR (Brain Dysfunction, Minimal)) OR (Dysfunction, Minimal Brain)) OR (Minimal Brain Dysfunction) |
| #10 | Search: ("Randomized Controlled Trial" [Publication Type]) OR (RCT) |
| #11 | #8 AND #9 AND #10 |

**Supplementary Table S3.** Definitions and Classification of Digital Interventions for ADHD.

| Interventions | | Definition | Typical software/platforms |
| --- | --- | --- | --- |
| Digital intervention | neurofeedback | A form of neuromodulation technology based on real-time brain activity monitoring, it guides individuals to consciously regulate specific neural activity patterns by converting their current brain state (typically in the form of electroencephalogram EEG or functional magnetic resonance imaging fMRI signals) into perceptible feedback signals (1) | EEGer、AFNI、Mensia |
|  | Computer cognitive tasks | Its core purpose is to scientifically evaluate or train specific cognitive functions, typically grounded in established neuropsychological theories, representing a digital adaptation of traditional paper-and-pencil tests (such as image classification, temporal selection, and origami tasks)(2) | BrainTrain、NeuroTrack |
|  | Computer Cognitive Games | Through the incorporation of gamification elements—such as instant feedback, progress rewards, and challenging levels—the aim is to enhance motivation, engagement, and training adherence (e.g., spot-the-difference games, fruit-cutting games, etc.) (3) | Braingame Brian、Cogoland |
|  | Mobile device games | Mobile device games, commonly referred to as ‘mobile games’ or ‘handheld games’, specifically denote gaming software designed to run on portable mobile devices such as smartphones and tablets(4) | AKL-T01、Tali Train |
|  | Mobile device video | Highly sensory-stimulating mobile videos provide children with ADHD with a transient “optimal arousal level” by modulating the brain's arousal state and activating specific neural circuits, thereby enhancing their attentional performance(5) | Ledongying、DingTalk |
|  | Virtual reality technology | Its core objective is to utilise devices such as head-mounted displays to immerse the user's vision, hearing and other senses within a world constructed entirely from digital elements, thereby creating an immersive sense of presence (6) | Dragon |

1. Tsuchiyagaito A, Misaki M, Kirlic N, Yu X, Sánchez SM, Cochran G, et al. Real-Time fMRI Functional Connectivity Neurofeedback Reducing Repetitive Negative Thinking in Depression: A Double-Blind, Randomized, Sham-Controlled Proof-of-Concept Trial. Psychother Psychosom. 2023;92(2):87-100.

2. McMurray J, Levy A, Pang W, Holyoke P. Psychometric Evaluation of a Tablet-Based Tool to Detect Mild Cognitive Impairment in Older Adults: Mixed Methods Study. J Med Internet Res. 2024;26:e56883.

3. Zhan J, Liu C, Wang Z, Cai Z, He J. Effects of game-based digital interventions for mental disorders: A meta-analysis. J Affect Disord. 2024;362:731-41.

4. Heilmann F, Formenti D, Trecroci A, Lautenbach F. The effects of a smartphone game training intervention on executive functions in youth soccer players: a randomized controlled study. Front Sports Act Living. 2023;5:1170738.

5. Choi Y, Lim JS, Choi H, Ryu YH, Seong E, Park I, et al. Narrative mobile video game-based cognitive training to enhance frontal function in patients with mild cognitive impairment. Sci Rep. 2025;15(1):195.

6. Bai Y, Luo Y, Goh CCW, Rui C, Gao R, Wu Y, et al. Relationships Between Parent Ratings of Attention-Deficit/Hyperactivity Disorder Behaviors and the Virtual Reality Attention Tracker in School-Aged Children: Cross-Sectional Study. J Med Internet Res. 2025;27:e76673.

# Supplementary Figures


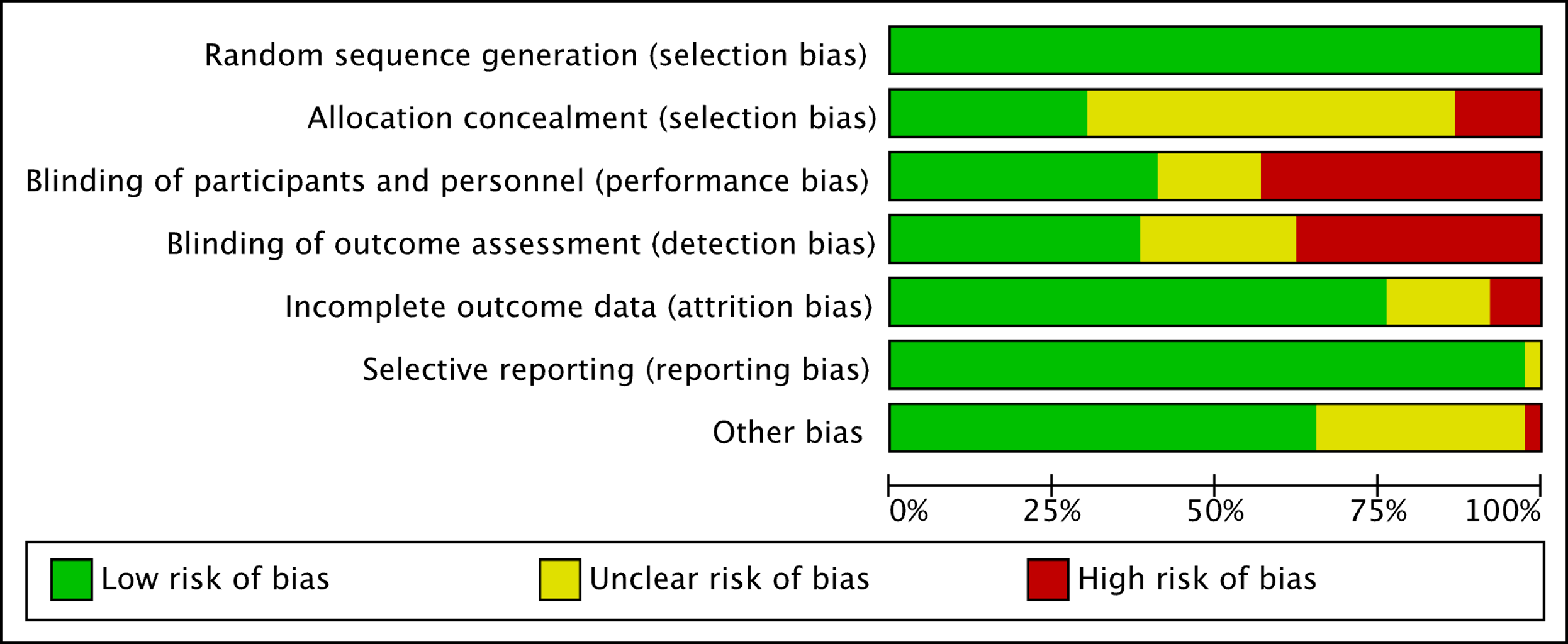


# Supplementary Figure S1. The overall risk of bias for all included studies.


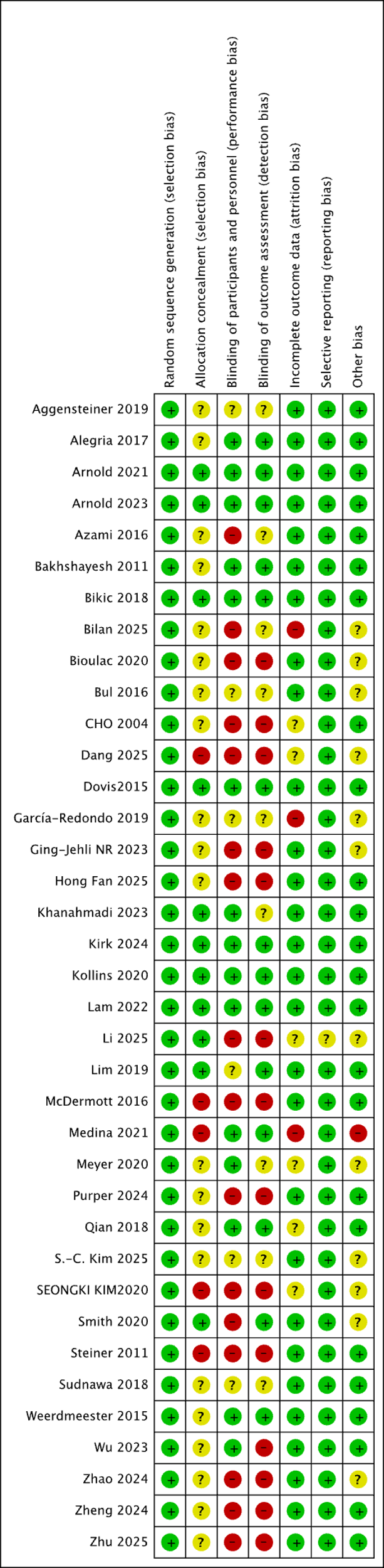


**Supplementary** **Figure S2.** The risk of bias for each study.


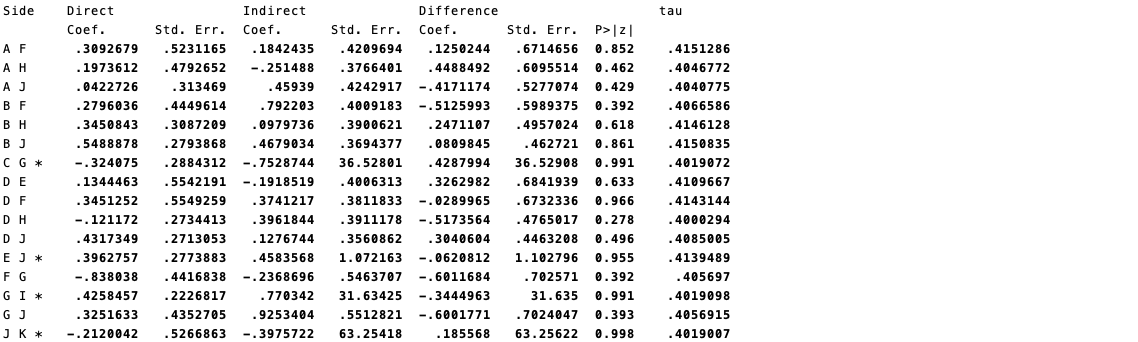


**Supplementary** **Figure S3.** Detection of Local Inconsistencies for inattention. A=Computer Cognitive Game, B=Computer Cognitive Task, C=Electromyographic Biofeedback, D=Mobile Device Game, E=Mobile Device Video, F=Medicinal Products, G=Neurofeedback H: Placebo-controlled, I: Simulated feedback, J: Usual Care, K: Virtual Reality Technology.


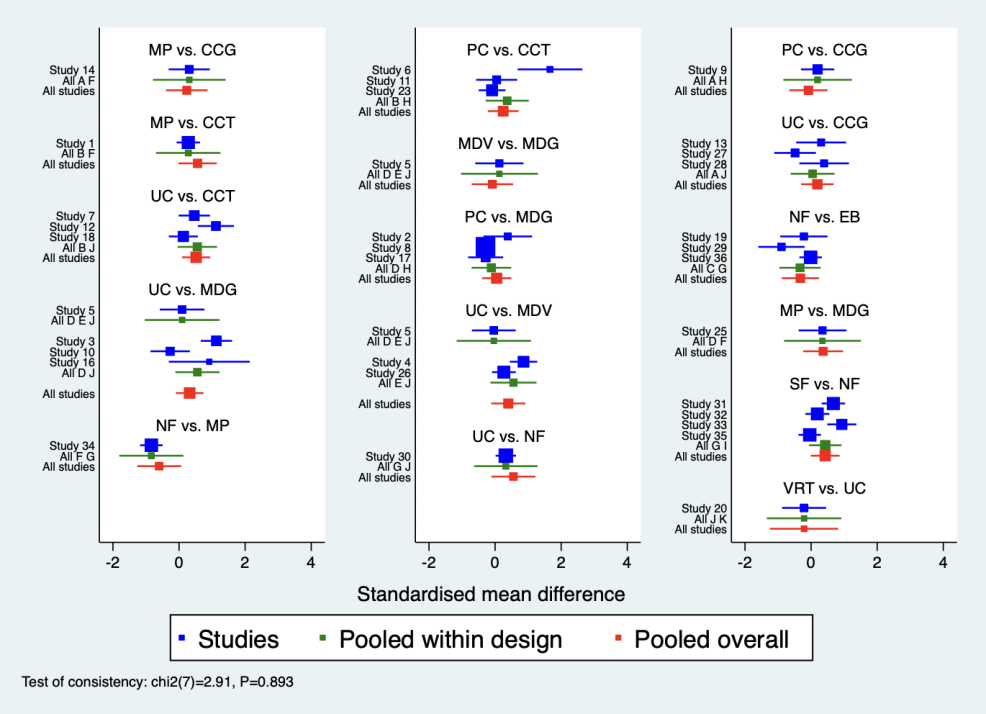


**Supplementary Figure S4.** Pairwise comparison of digital cognitive interventions for inattention. NF: Neurofeedback; CCT: Compute Cognitive Task; CCG: Computer Cognitive Game; MDG: Mobile Device Game; VRT: Virtual Reality Technology; MDV: Mobile Device Video; PC: Placebo-controlled; UC: Usual Care SF: Simulated feedback MP: Medicinal Products; EB: Electromyographic Biofeedback.


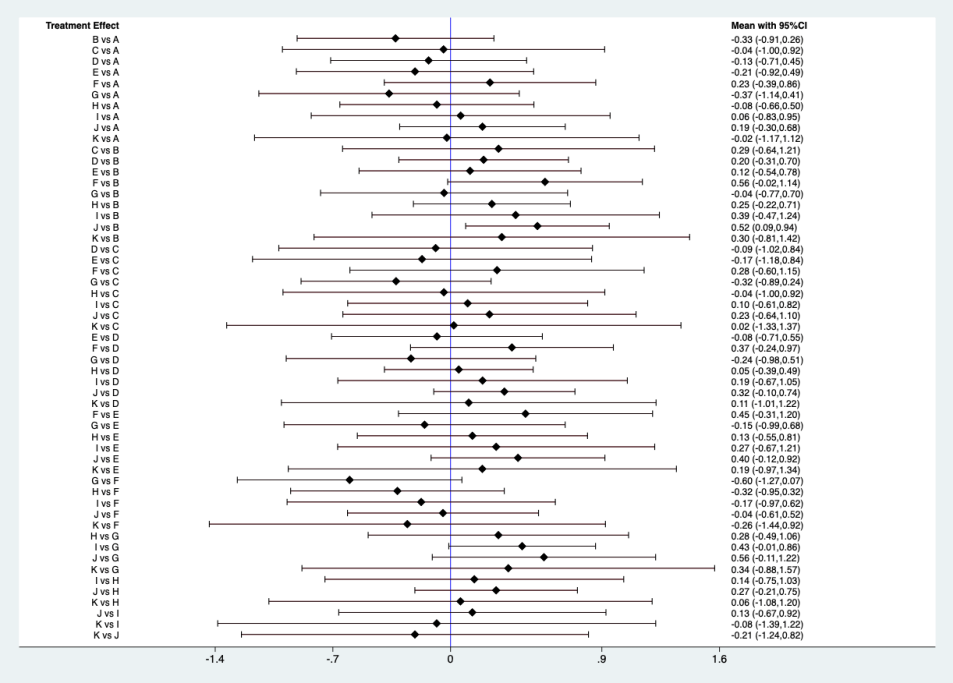


**Supplementary Figure S5.** Interval Plot for the Network Meta-Analysis of inattention. A=Computer Cognitive Game, B=Computer Cognitive Task, C=Electromyographic Biofeedback, D=Mobile Device Game, E=Mobile Device Video, F=Medicinal Products, G=Neurofeedback H: Placebo-controlled, I: Simulated feedback, J: Usual Care, K: Virtual Reality Technology


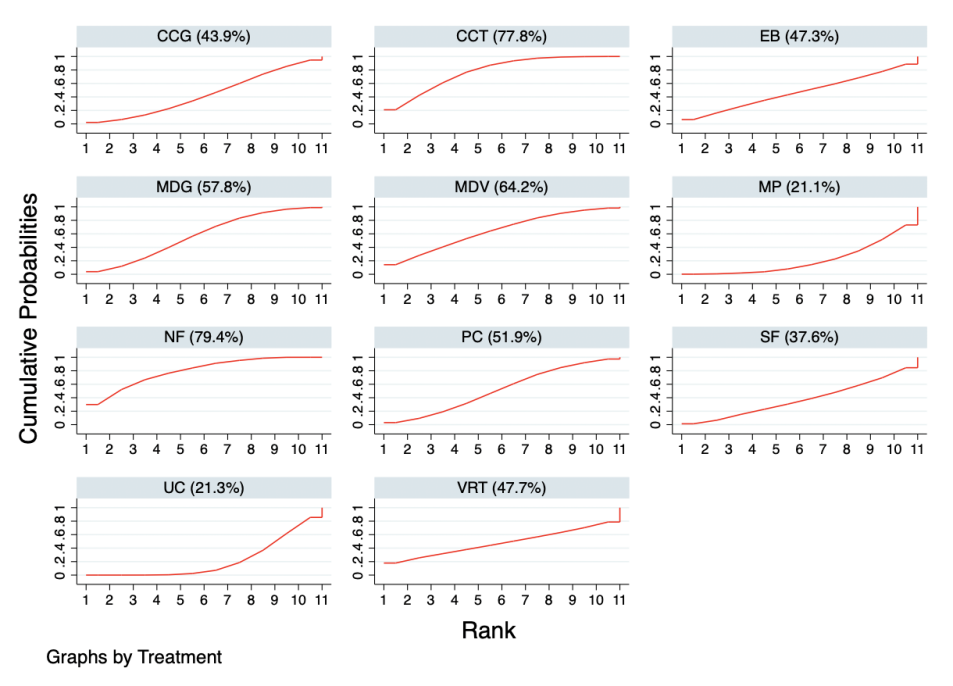


**Supplementary Figure S6.** Cumulative ranking probability plots for inattention. The horizontal axis represents the possible rank of each treatment (from best to worst according to the outcome). The vertical axis represents the cumulative probability for each treatment to be the best option, the best of 2 options, the best of 3 options, and so on. NF: Neurofeedback; CCT: Compute Cognitive Task; CCG: Computer Cognitive Game; MDG: Mobile Device Game; VRT: Virtual Reality Technology; MDV: Mobile Device Video; PC: Placebo-controlled; UC: Usual Care; SF: Simulated feedback MP: Medicinal Products; EB: Electromyographic Biofeedback.


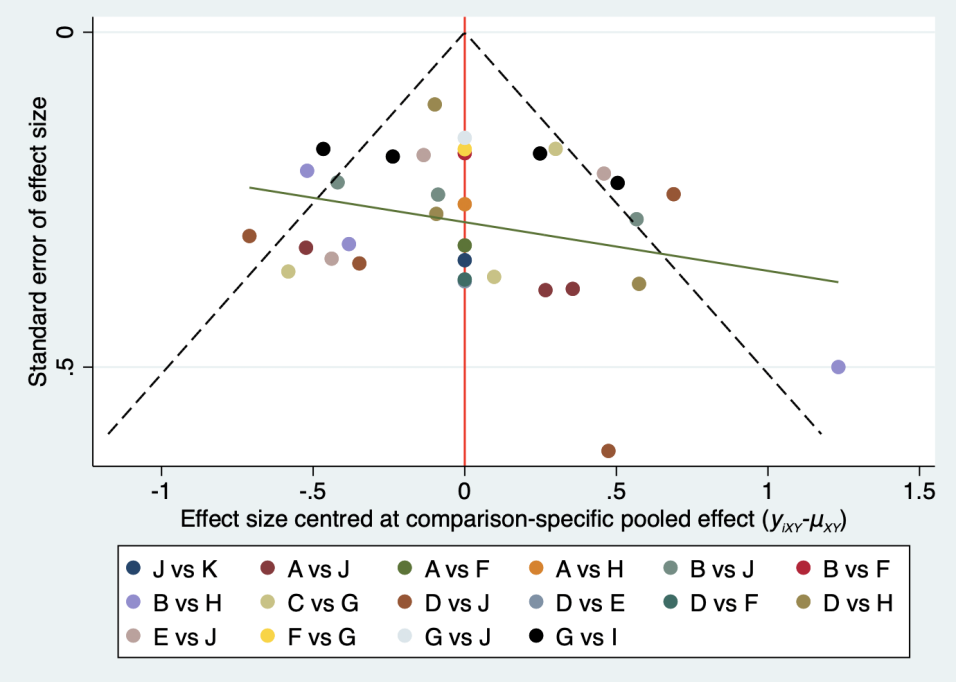


**Supplementary Figure S7.** Funnel plot of included literature for inattention. A=Computer Cognitive Game, B=Computer Cognitive Task, C=Electromyographic Biofeedback, D=Mobile Device Game, E=Mobile Device Video, F=Medicinal Products, G=Neurofeedback, H: Placebo-controlled, I: Simulated feedback, J: Usual Care, K: Virtual Reality Technology.


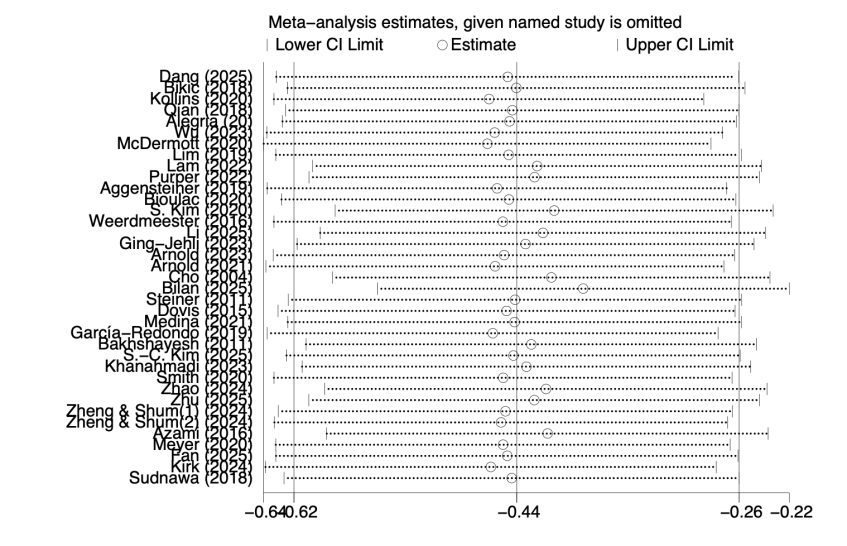


**Supplementary Figure S8.** Sensitivity Analysis Chart for Attention Deficit.


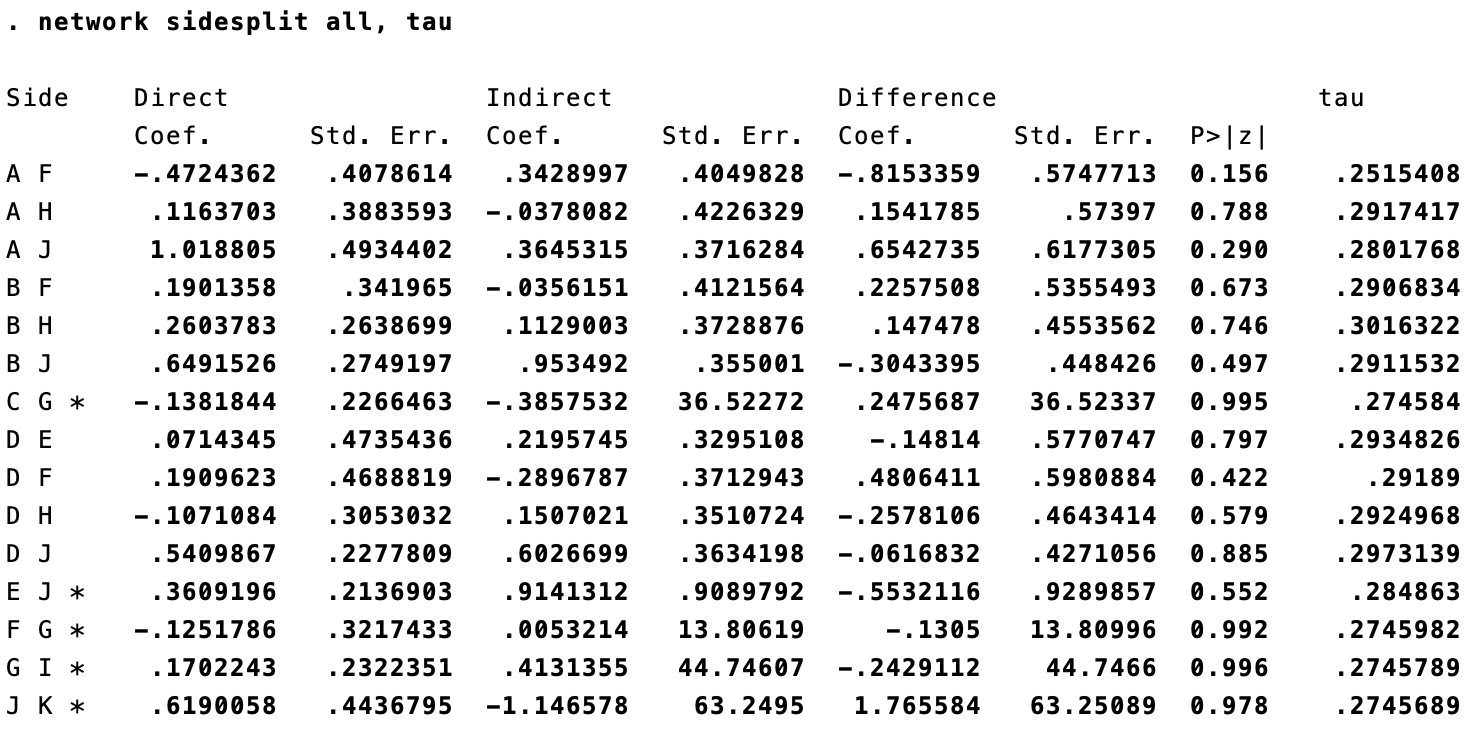


**Supplementary Figure S9.** Detection of Local Inconsistencies for hyperactive impulse. A=Computer Cognitive Game, B=Computer Cognitive Task, C=Electromyographic Biofeedback, D=Mobile Device Game, E=Mobile Device Video, F=Medicinal Products, G=Neurofeedback H: Placebo-controlled, I: Simulated feedback, J: Usual Care, K: Virtual Reality Technology.


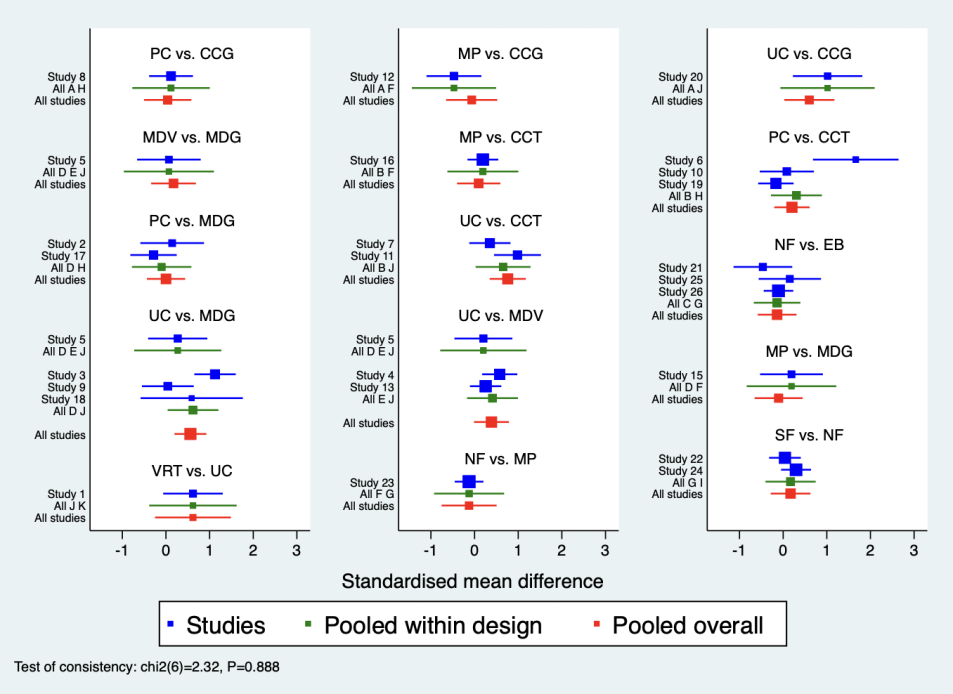


**Supplementary Figure S10.** Pairwise comparison of digital cognitive interventions for hyperactive impulse. NF: Neurofeedback; CCT: Compute Cognitive Task; CCG: Computer Cognitive Game; MDG: Mobile Device Game; VRT: Virtual Reality Technology; MDV: Mobile Device Video; PC: Placebo-controlled; UC: Usual Care SF: Simulated feedback MP: Medicinal Products; EB: Electromyographic Biofeedback.


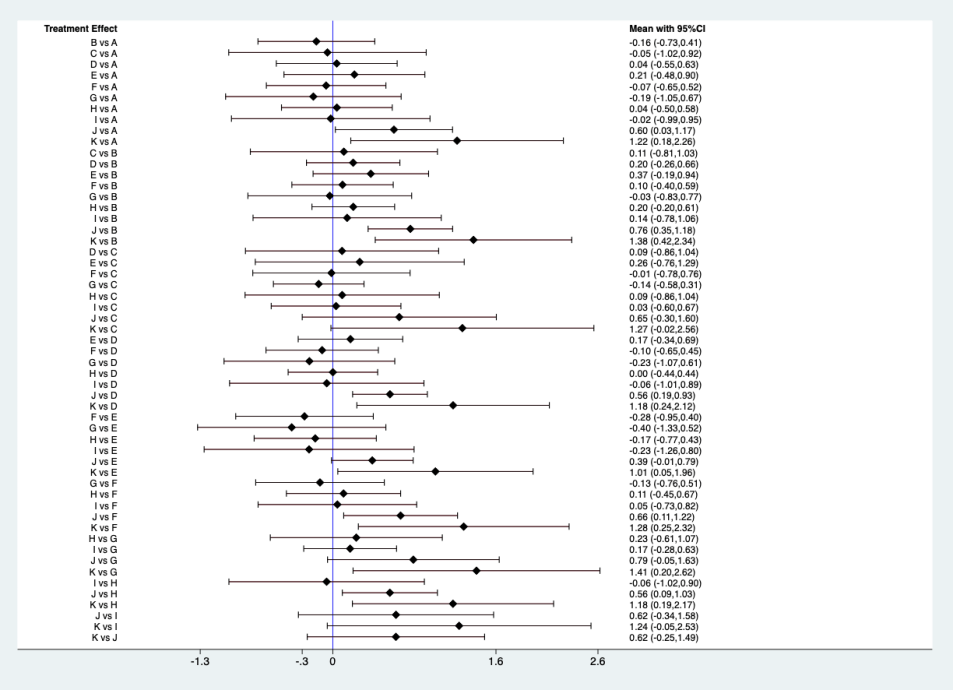


**Supplementary Figure S11.** Interval Plot for the Network Meta-Analysis of hyperactive impulse. A=Computer Cognitive Game, B=Computer Cognitive Task, C=Electromyographic Biofeedback, D=Mobile Device Game, E=Mobile Device Video, F=Medicinal Products, G=Neurofeedback H: Placebo-controlled, I: Simulated feedback, J: Usual Care, K: Virtual Reality Technology.

**
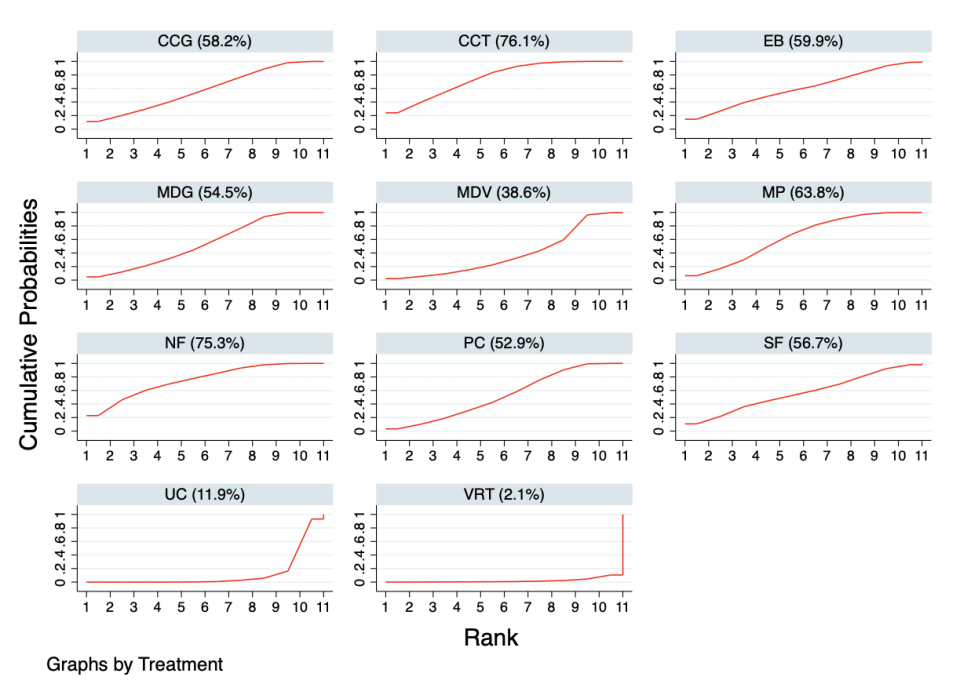
**

**Supplementary Figure S12.** Cumulative ranking probability plots for hyperactive impulse. The horizontal axis represents the possible rank of each treatment (from best to worst according to the outcome). The vertical axis represents the cumulative probability for each treatment to be the best option, the best of 2 options, the best of 3 options, and so on.NF: Neurofeedback; CCT: Compute Cognitive Task; CCG: Computer Cognitive Game;MDG: Mobile Device Game; VRT: Virtual Reality Technology; MDV: Mobile Device Video; PC:Placebo-controlled; UC:Usual Care; SF:Simulated feedback MP:Medicinal Products; EB:Electromyographic Biofeedback.


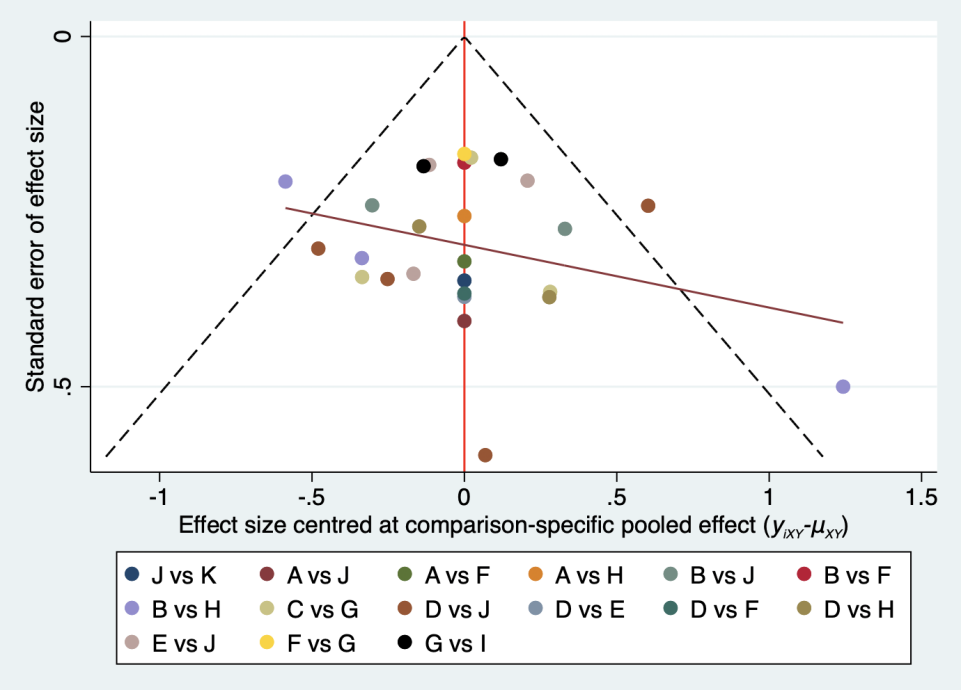


**Supplementary Figure S13** Funnel plot of included literature for hyperactive impulse. A=Computer Cognitive Game, B=Computer Cognitive Task, C=Electromyographic Biofeedback, D=Mobile Device Game, E=Mobile Device Video, F=Medicinal Products, G=Neurofeedback H: Placebo-controlled, I: Simulated feedback, J: Usual Care, K: Virtual Reality Technology.


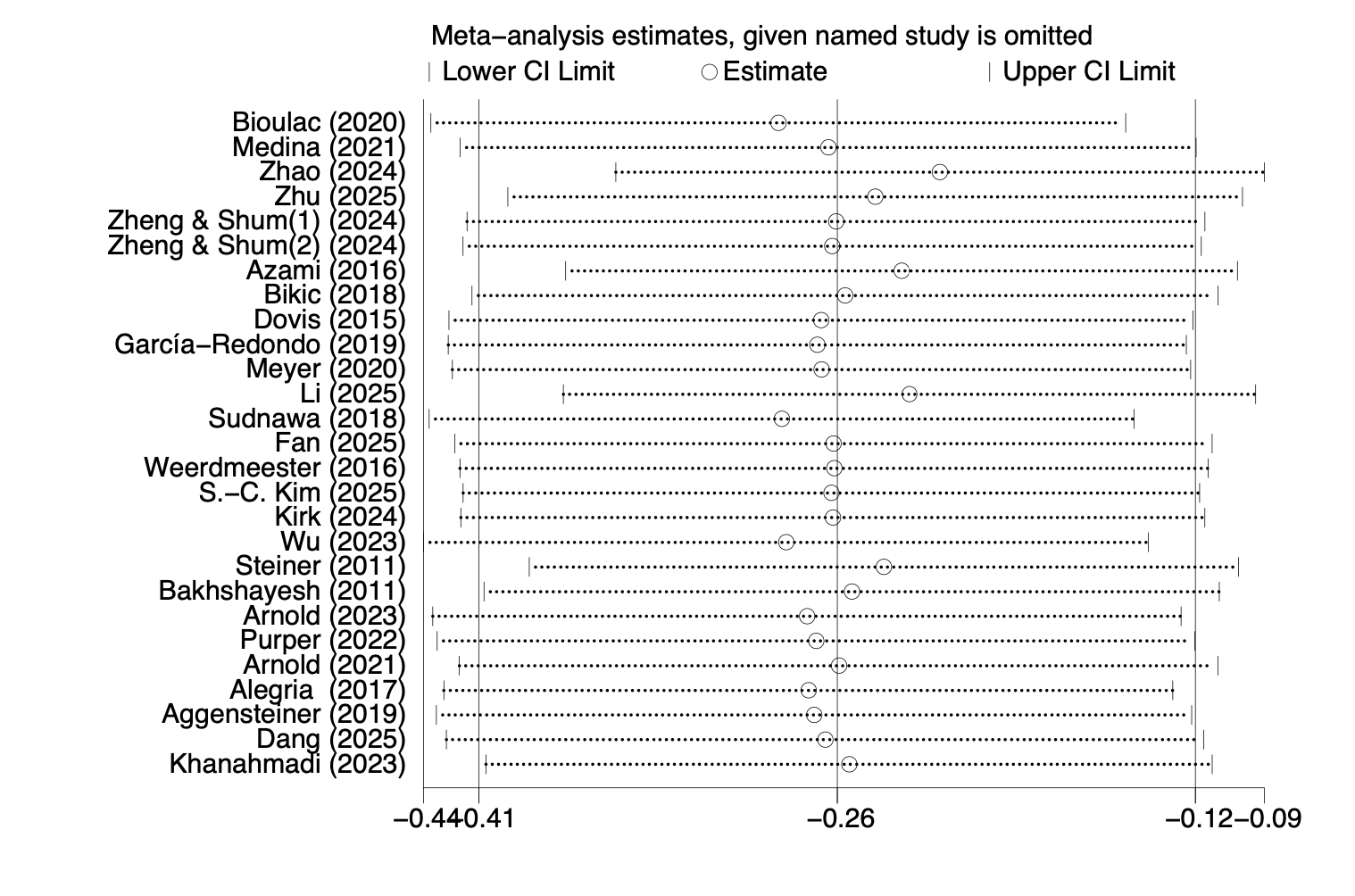


**Supplementary Figure S14.** Sensitivity Analysis Chart for Hyperactive Impulsivity.


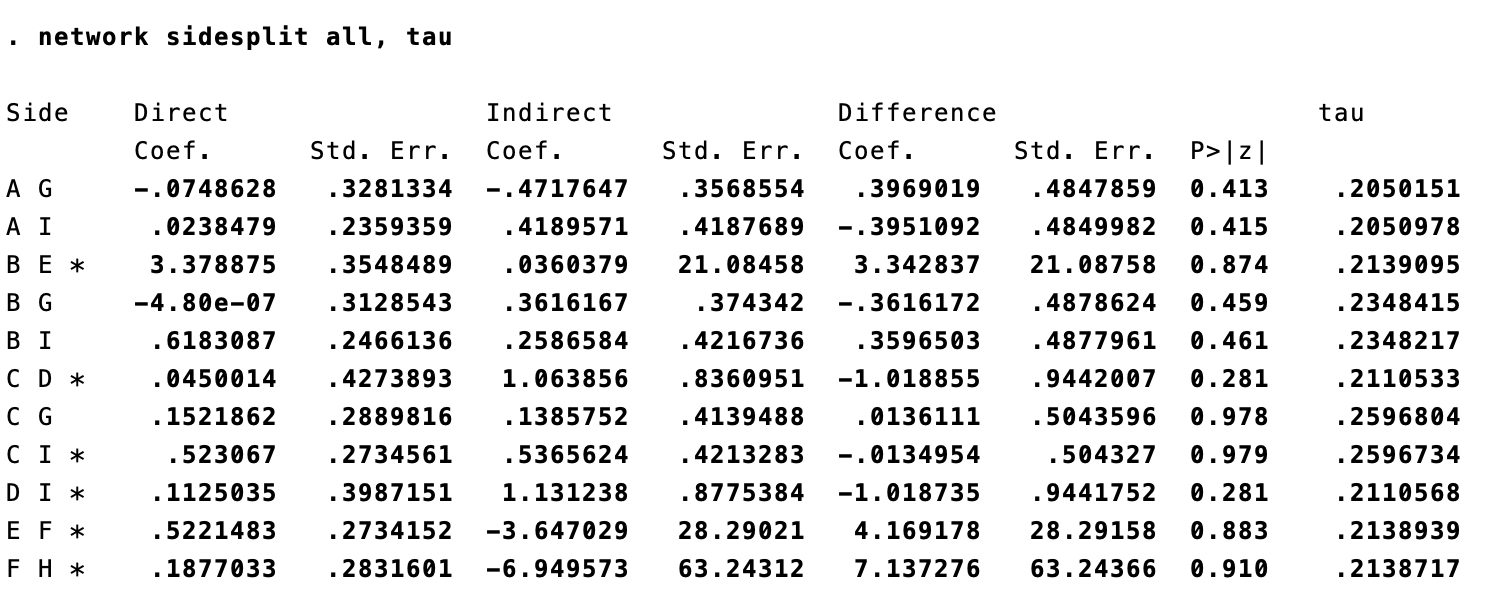


**Supplementary Figure S15.** Detection of Local Inconsistencies for executive function. A=Computer Cognitive Game, B=Computer Cognitive Task, C=Mobile Device Game, D=Mobile Device Video, E=Medicinal Products, F=Neurofeedback, G: Placebo-controlled, H: Simulated feedback, I: Usual Care.


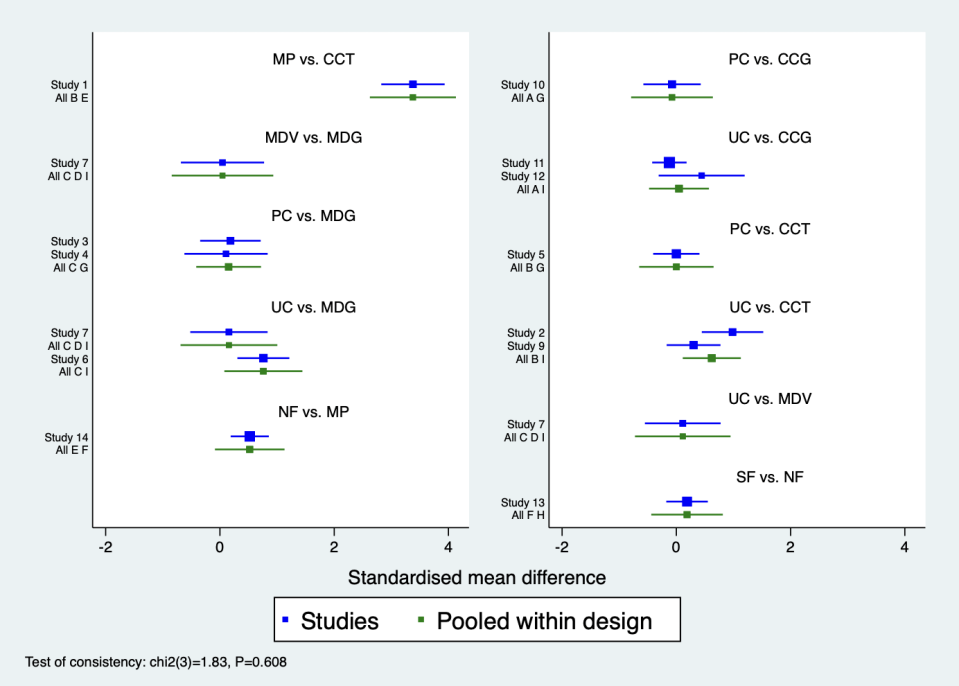


**Supplementary Figure S16.** Pairwise comparison of digital cognitive interventions for executive function. NF: Neurofeedback; CCT: Compute Cognitive Task; CCG: Computer Cognitive Game; MDG: Mobile Device Game; MDV: Mobile Device Video; PC: Placebo-controlled; UC: Usual Care SF: Simulated feedback MP: Medicinal Products.


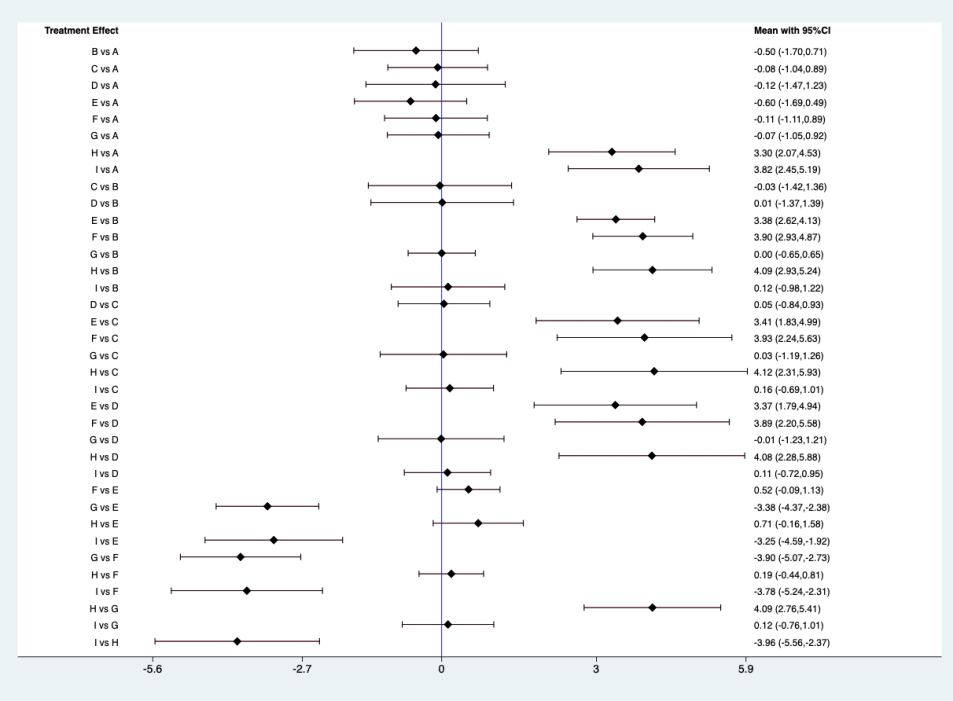


**Supplementary Figure S17.** Interval Plot for the Network Meta-Analysis of executive function. A=Computer Cognitive Game, B=Computer Cognitive Task, C=Mobile Device Game, D=Mobile Device Video, E=Medicinal Products, F=Neurofeedback, G: Placebo-controlled, H: Simulated feedback, I: Usual Care.


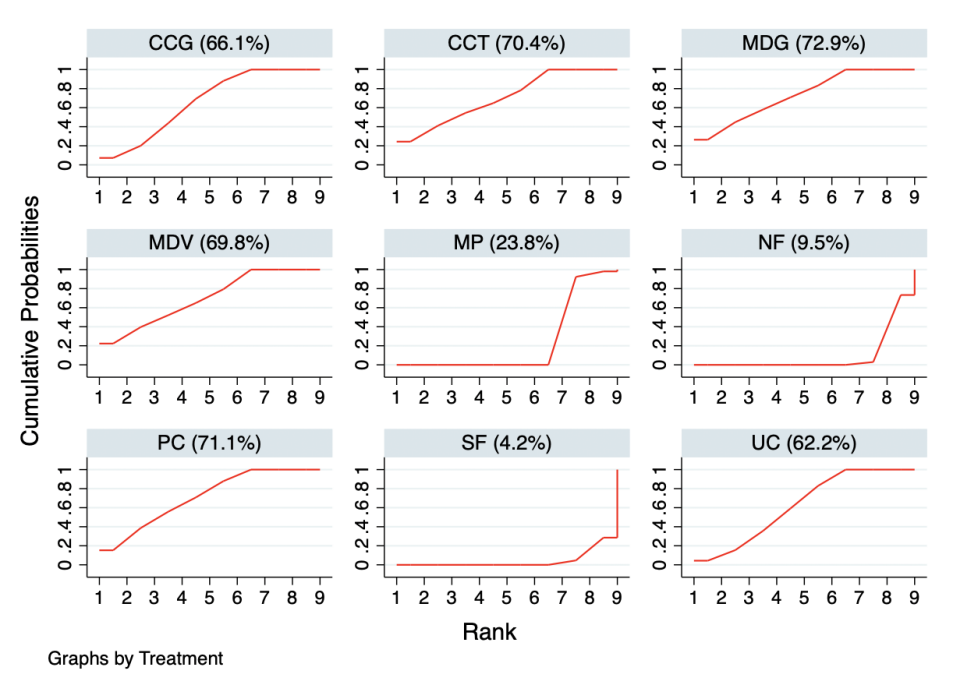


**Supplementary Figure S18** Cumulative ranking probability plots for executive function. The horizontal axis represents the possible rank of each treatment (from best to worst according to the outcome). The vertical axis represents the cumulative probability for each treatment to be the best option, the best of 2 options, the best of 3 options, and so on. NF: Neurofeedback; CCT: Compute Cognitive Task; CCG: Computer Cognitive Game; MDG: Mobile Device Game; MDV: Mobile Device Video; PC: Placebo-controlled; UC: Usual Care; SF :Simulated feedback MP: Medicinal Products.


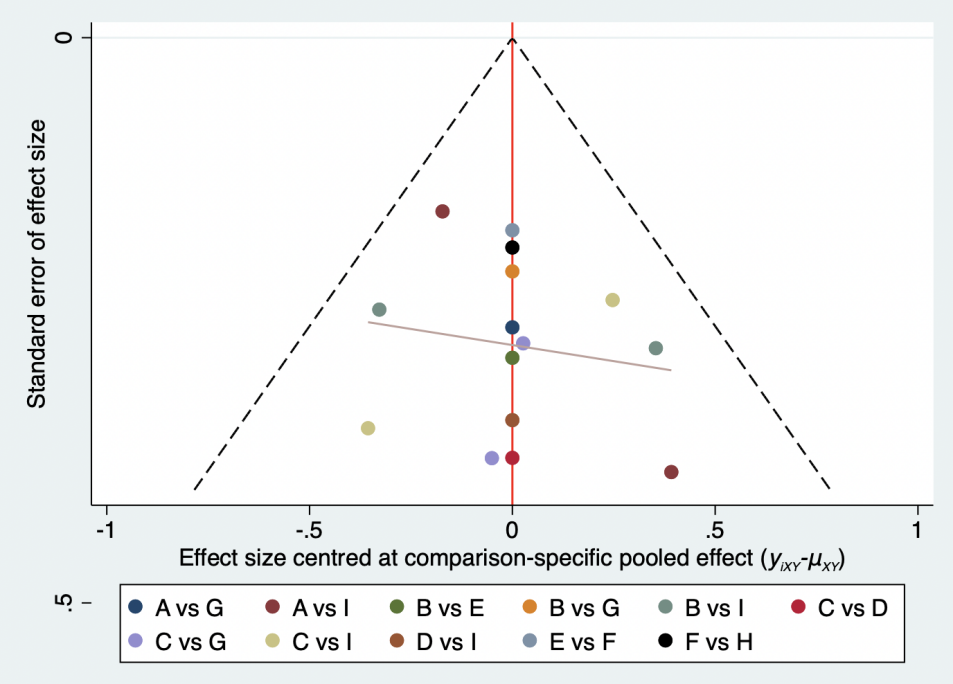


**Supplementary Figure S19.** Funnel plot of included literature for executive function. A=Computer Cognitive Game, B=Computer Cognitive Task, C=Mobile Device Game, D=Mobile Device Video, E=Medicinal Products, F=Neurofeedback, G: Placebo-controlled, H:Simulated feedback, I: Usual Care.

**
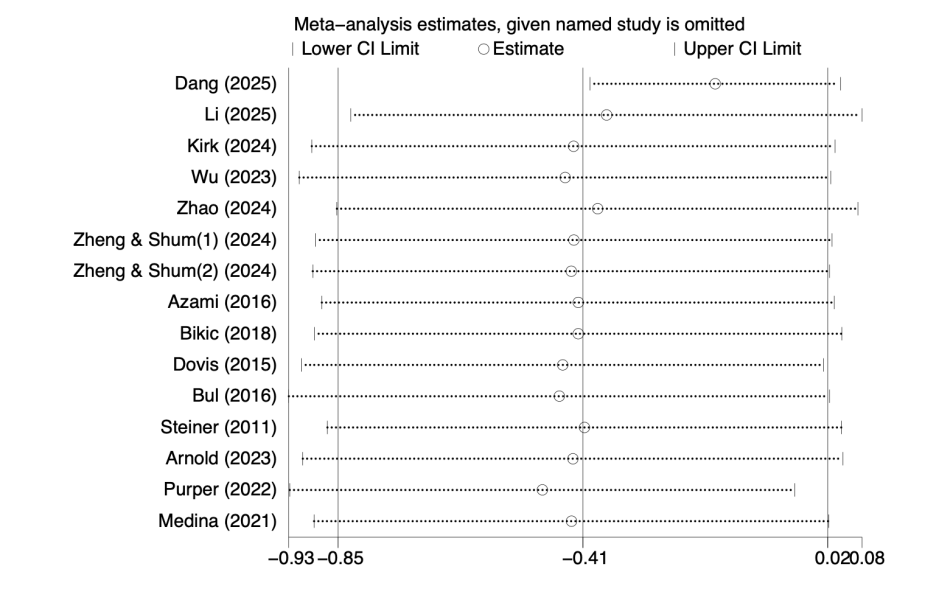
**

**Supplementary Figure S20.** Sensitivity Analysis Chart for Executive Functioning.

**
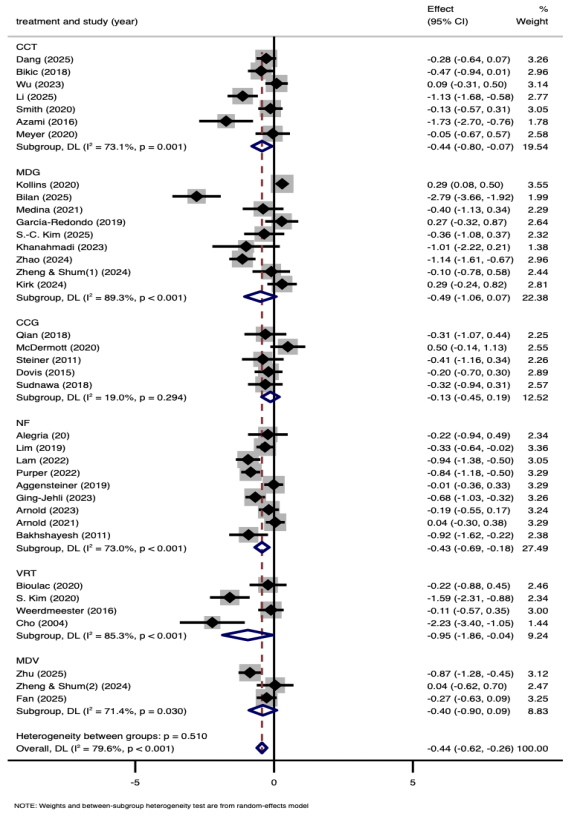
**

**Supplementary Figure S21.** Meta-analysis forest plot for subgroup analysis of intervention measures.

**
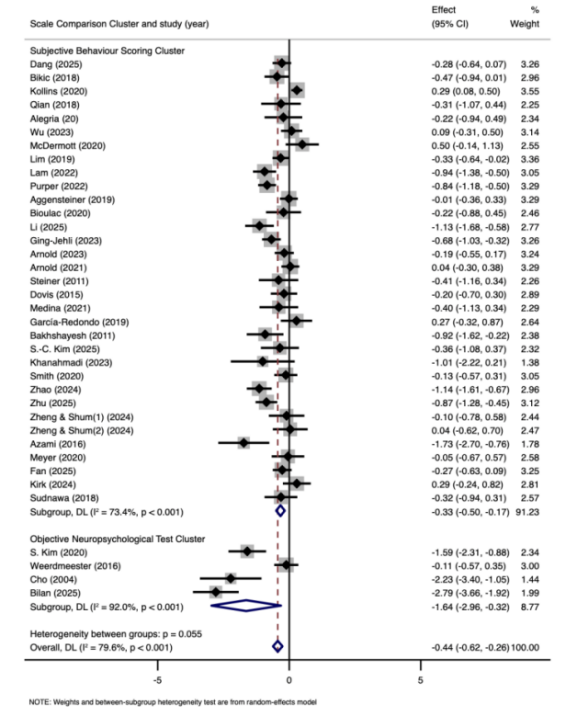
**

**Supplementary Figure S22.** Meta-analysis forest plot for subgroup analysis of Scale Comparison Cluster.

**
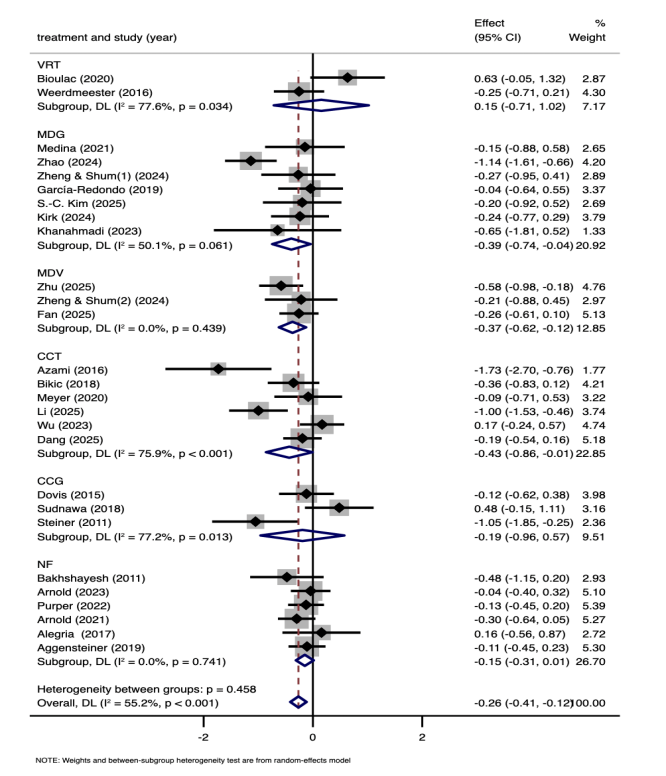
**

**Supplementary Figure S23** Meta-analysis forest plot for subgroup analysis of intervention measures.


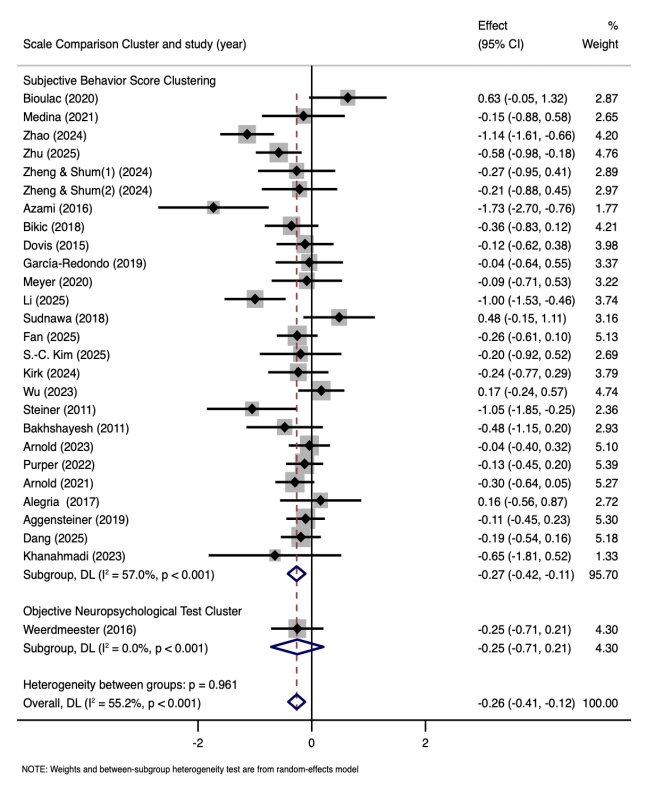


**Supplementary Figure S24.** Meta-analysis forest plot for subgroup analysis of Scale Comparison Cluster.

**
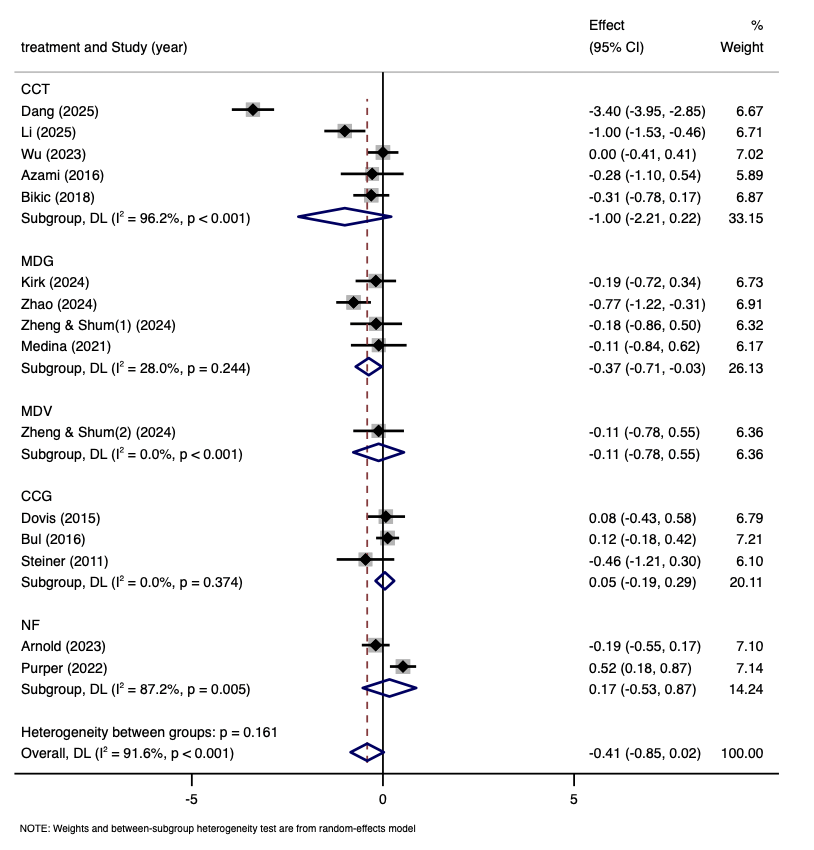
**

**Supplementary Figure S25.** Meta-analysis forest plot for subgroup analysis of intervention measures.

**
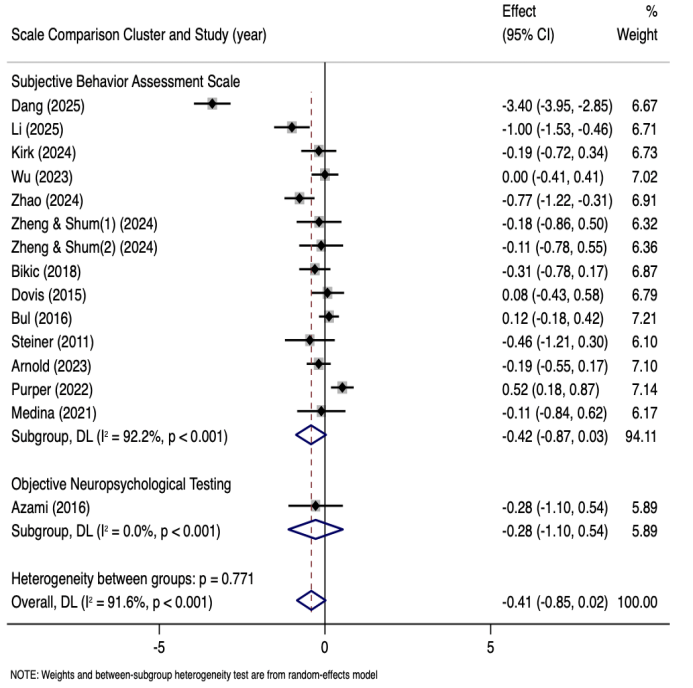
**

**Supplementary Figure S26.** Meta-analysis forest plot for subgroup analysis of Scale Comparison Cluster.
